# Identifying key factors for predicting O6-Methylguanine-DNA methyltransferase status in adult patients with diffuse glioma: a multimodal analysis of demographics, radiomics, and MRI by variable Vision Transformer

**DOI:** 10.1007/s00234-024-03329-8

**Published:** 2024-03-12

**Authors:** Takuma Usuzaki, Kengo Takahashi, Ryusei Inamori, Yohei Morishita, Takashi Shizukuishi, Hidenobu Takagi, Mami Ishikuro, Taku Obara, Kei Takase

**Affiliations:** 1 Department of Diagnostic Radiology, Tohoku University Hospital, 1-1 Seiryo-machi, Aoba-ku, Sendai, Miyagi, Miyagi 980-8574 Japan; 2 Tohoku University Graduate School of Medicine, 2-1 Seiryo-machi, Aoba-ku, Sendai, Miyagi, Miyagi 980-8573 Japan; 3 Department of Advanced MRI Collaborative Research, Graduate School of Medicine, Tohoku University, 2-1 Seiryo-machi, Aoba-ku, Sendai, Miyagi, Miyagi 980-8573 Japan; 4 Tohoku University Graduate School of Medicine, Division of Molecular Epidemiology, 2-1 Seiryo-machi, Aoba-ku, Sendai, Miyagi, Miyagi 980-8573 Japan; 5 Tohoku University Graduate School of Medicine, Division of Molecular Epidemiology, Department of Preventive Medicine and Epidemiology, Tohoku Medical Megabank Organization, 2-1 Seiryo-machi, Aoba-ku, Sendai, Miyagi, Miyagi 980-8573 Japan; 6 Tohoku University Hospital, Department of Pharmaceutical Sciences, 1-1 Seiryo-machi, Aoba-ku, Sendai, Miyagi, Miyagi 980-8574 Japan

**Keywords:** O6-methylguanine-DNA methyl transferase (MGMT), glioma, deep learning, vision transformer (ViT), variable vision transformer (vViT)

## Abstract

**Purpose:**

This study aimed to perform multimodal analysis by vision transformer (vViT) in predicting O6-methylguanine-DNA methyl transferase (MGMT) promoter status among adult patients with diffuse glioma using demographics (sex and age), radiomic features, and MRI.

**Methods:**

The training and test datasets contained 122 patients with 1,570 images and 30 patients with 484 images, respectively. The radiomic features were extracted from enhancing tumors (ET), necrotic tumor cores (NCR), and the peritumoral edematous/infiltrated tissues (ED) using contrast-enhanced T1-weighted images (CE-T1WI) and T2-weighted images (T2WI). The vViT had 9 sectors; 1 demographic sector, 6 radiomic sectors (CE-T1WI ET, CE-T1WI NCR, CE-T1WI ED, T2WI ET, T2WI NCR, and T2WI ED), 2 image sectors (CE-T1WI, and T2WI). Accuracy and area under the curve of receiver-operating characteristics (AUC-ROC) were calculated for the test dataset. The performance of vViT was compared with AlexNet, GoogleNet, VGG16, and ResNet by McNemar and Delong test. Permutation importance (PI) analysis with the Mann–Whitney U test was performed.

**Results:**

The accuracy was 0.833 (95% confidence interval [95%CI]: 0.714–0.877) and the area under the curve of receiver-operating characteristics was 0.840 (0.650–0.995) in the patient-based analysis. The vViT had higher accuracy than VGG16 and ResNet, and had higher AUC-ROC than GoogleNet (*p*<0.05). The ED radiomic features extracted from the T2-weighted image demonstrated the highest importance (PI=0.239, 95%CI: 0.237–0.240) among all other sectors (*p*<0.0001).

**Conclusion:**

The vViT is a competent deep learning model in predicting MGMT status. The ED radiomic features of the T2-weighted image demonstrated the most dominant contribution.

**Supplementary Information:**

The online version contains supplementary material available at 10.1007/s00234-024-03329-8.

## Introduction

Glioma is one of the most common primary tumors of the central nervous system (CNS) [1, 2]. The 2021 World Health Organization (WHO) CNS tumors classification recommends performing CNS tumor grading by adding molecular parameters to histological features [3, 4] because certain molecular markers can provide prognostic information [3]. Over the past decade, the methylation status of O6-methylguanine-DNA methyl transferase (MGMT) promoter is associated with an overall survival rate as well as their diagnostic value [5–7]. When patients with glioblastoma received chemotherapy, patients with methylation of MGMT promoter results in longer survival compared to patients with unmethylated MGMT promoter [8, 9]. MGMT promoter methylation was strongly associated with a superior progression-free rate and survival rate at 12 months [10]. A biopsy and histological examination need to be performed to determine MGMT promoter methylation. The brain tumor property is evaluated by radiological imaging when a biopsy cannot be performed due to reasons, such as tumor size, tumor location, patient comorbidity, and patient condition [10, 11]. Recent studies aimed to predict MGMT promoter methylation by analyzing radiomic features or images using machine learning algorithms, including a convolutional neural network (CNN) [12–21]. However, the most dominant factors among patient characteristics, radiomic features, and magnetic resonance imaging (MRI) for predicting MGMT promoter methylation remain unclear. This is partly because machine and deep learning have a limitation in simultaneously analyzing these factors in one model [22]. Identifying the dominant factor to predict MGMT promoter methylation is important in the context of the global development of multimodal artificial intelligence solutions [23, 24].

A previous study has proposed a Vision Transformer (ViT)-inspired model, named variable ViT (vViT) that analyzes multiple sequences of different lengths [22, 25–27]. The vViT simultaneously handles multimodal factors (patient characteristics, radiomic features, and MRI), calculating prediction accuracy for each factor and then integrating them into the overall performance. One strength of vViT is its ability to quantitatively evaluate, or identify, the most dominant factor by calculating the prediction accuracy for each factor in a single model [27]. This strength is attributed to that the vViT analyzes input factors separately. However, limited studies have applied vViT to predicting MGMT promoter methylation among adult patients with diffuse gliomas. This study aimed to investigate the performance of vViT and identify the dominant factor among patient characteristics, radiomic features, and MRI using vViT in predicting MGMT promoter methylation among adult patients with diffuse glioma.

## Material and methods

### Data collection

This cross-sectional study obtained all data from the University of California San Francisco Preoperative Diffuse Glioma MRI (UCSF-PDGM) dataset following the Cancer Image Archive data usage policy and restrictions [28, 29]. The UCSF institutional review board approved data collection, with a waiver for consent, and retrospectively performed this study following the relevant guidelines and regulations. The UCSF-PDGM dataset consisted of 501 adult patients with histopathologically confirmed diffuse glioma (following the 2021 WHO classification) who underwent preoperative MRI, initial tumor resection, and tumor genetic testing at a single medical center from 2015 to 2021 [4]. The MGMT promoter methylation of tumors was tested by immunohistochemical staining, Sanger, or next-generation genetic sequencing in the UCSF-PDGM dataset [28].

### Radiomic feature extraction and image processing

Image data in the UCSF-PDGM dataset first underwent automated segmentation using an ensemble model consisting of brain tumor segmentation challenge algorithms [28, 30]. The segmentation was finally approved by a board-certified neuro-radiologist with more than 15 years of experience [28, 30]. Segmentation included three major tumor compartments: enhancing tumor (ET), necrotic tumor core (NCR), and the peritumoral edematous/infiltrated tissue (ED). We extracted 105 radiomic features (Appendix 1) from each compartment of contrast material-enhanced T1-weighted images (CE-T1WI) and T2-weighted images (T2WI) using the PyRadiomics package [31]. We excluded images in which the annotation image contained fewer than 256 pixels from the extraction of radiomic features. After extracting the radiomic features, the image was cropped to the minimum rectangle that contained the tumor. The cropped image was expanded to a 128 × 128 image using the Python Pillow package with the LANCZOS option.

### Datasets construction and vViT setting

The construction of the model excluded patients (i) with no age or sex information, (ii) with unexplained tumor MGMT promoter methylation, (iii) with radiomic features of ET, NCR, or ED that could not be extracted from CE-T1WI and T2WI images, and (iv) with images far from the standard deviation of the mean number of images. Additionally, (v) random selection was conducted to equalize the number of MGMT-methylated images to that of MGMT-unmethylated images. The criterion (v) was imposed to perform image-based analysis in the balanced setting and to avoid overestimation of performance. After imposing the UCSF-PDGM dataset criteria, 152 patients with 2,054 images (1,027 MGMT-methylated, 1,027 MGMT unmethylated) remained (Appendix 2). These images were randomly categorized into a training dataset (122 patients with 1,570 images [785 MGMT-methylated and 785 MGMT unmethylated]) and a test dataset (30 patients with 484 images [242 MGMT-methylated and 242 MGMT unmethylated]). All patients in the training and test datasets had grade 4 glioma. We performed an analysis of variance (ANOVA) to select the radiomic features associated with MGMT promoter methylation. The highest 64 radiomic features in F-value were selected in decreasing order from each ET, NCR, and ED of the training datasets. The selected features were shown in Appnedix 3 with F-score and p-value.

**Fig. 1:**
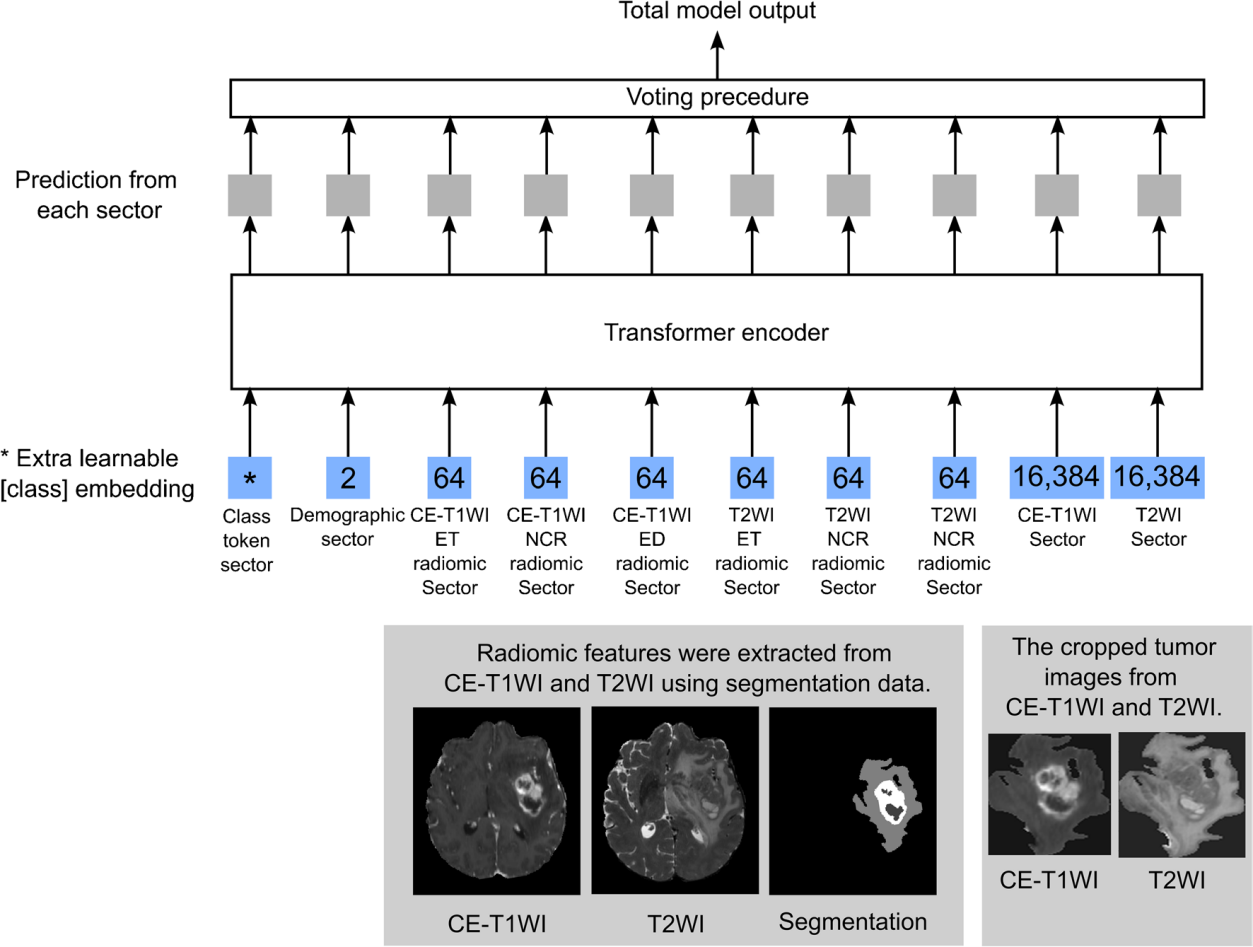
Scheme of the variable vision transformer (vViT) constructed in this study. The number in the blue rectangle represents the length of arrays input into vViT; demographics (2 features; age and sex), CE-T1WI ET (64 radiomic features), CE-T1WI NCR (64 radiomic features), CE-T1WI ED (64 radiomic features), T2WI ET (64 radiomic features), T2WI NCR (64 radiomic features), T2WI ED (64 radiomic features) radiomic, CE-T1WI image, and T2WI image sectors. All arrays were converted to 1-dimensional arrays before inputting to vViT. The gray rectangle represents the prediction from each sector. The total model output was obtained by voting. Based on the original vViT we attached the class token sector. Abbreviations: CE-T1WI: contrast-enhanced T1-weighted image; T2WI: T2-weighted image; ET: enhancing tumor; NCR: necrotic tumor core; ED: peritumoral edematous/infiltrated tissue

Figure 1 shows an architectural overview of the vViT constructed in the present study. The constructed vViT demonstrated nine sectors, including the class token sector: demographics, CE-T1WI ET, CE-T1WI NCR, CE-T1WI ED, T2WI ET, T2WI NCR, T2WI ED radiomic, CE-T1WI image, and T2WI image sectors. All data was converted to 1-dimensional arrays before inputting into vViT. The prediction of MGMT methylation from each sector can be individually derived. The performance of each sector can be calculated. The prediction of each sector was integrated by voting: among predictions from nine sectors, the majority prediction was regarded as the total model output. Pytorch version 1.7.1 was used to implement vViT as the deep learning framework. Binary cross entropy was optimized by the Adam optimizer (β1=0.9, β2=0.999, ε=1.0×10^–8^, weight-decay=0, and AMSGrad=False). The detailed explanation of vViT and terminologies including Binary cross entropy and Adam optimizer are written in Appendix 4.

### Statistical analysis

Patient characteristics and calculated values are shown as means and 95% confidence interval (95%CI) or as numbers (n) and ratios (%). We calculated classification accuracy, sensitivity, specificity, positive predictive value (PPV), negative predictive value (NPV), F-score, the area under the curve of receiver-operating characteristics (AUC-ROC), logarithmic loss, and Cohen’s κ coefficient as metrics to evaluate the performance of vViT. We organized the output of vViT to the prediction of each patient (patient-based analysis) because vViT was implemented to calculate metrics for each image (image-based analysis). This organization used voting and mean for binary and continuous variables, respectively. The performance of CE-T1WI and T2WI image sectors in vViT was compared with AlexNet [32], GoogleNet [33], visual geometry group (VGG) 16 [34], and ResNet [35]. These CNN models were tested after 1000 epochs training using each CE-T1WI and T2WI images. McNemar test and DeLong test were performed to compare contingency table and AUC-ROC, respectively. Permutation importance analysis was performed to evaluate the contribution from each sector to output. The following three calculation steps were performed to evaluate permutation importance.

(i) Permutation was performed in the respective sector following the previously reported method [36]. This implementation assigned the data of a patient to another patient.
(ii) After permutation, the accuracy was calculated using trained vViT.
(iii) The difference between the original accuracy and the accuracy calculated using the permutated dataset was then saved. We defined the difference as permutation importance.

**Fig. 2:**
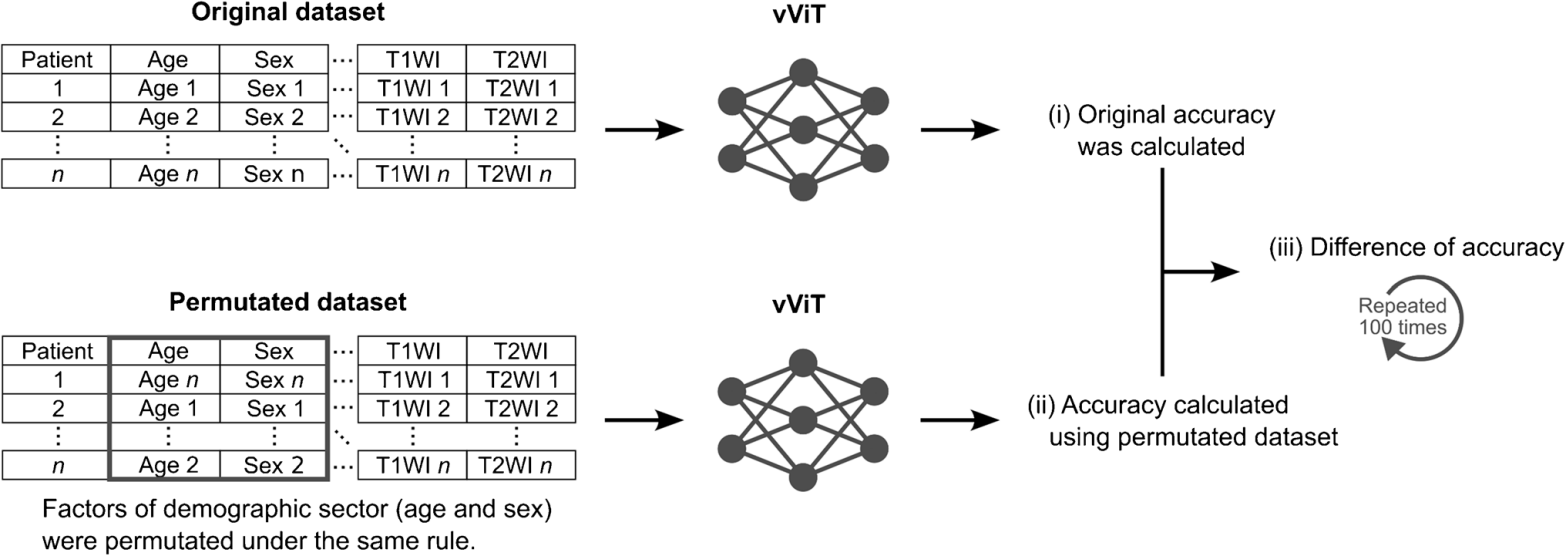
Procedures of permutation importance analysis. Demographics (age and sex) were permutated as an example. The following three steps were performed. (i) The original accuracy was calculated using the original dataset and trained variable vision transformer (vViT). (ii) Permutation was then performed, and accuracy was calculated using a permutated dataset and trained vViT. (iii) The difference between the original accuracy and the accuracy calculated using a permutated dataset was calculated. We defined the difference as the permutation importance. These processes were repeated 100 times by changing the permutation pattern.

Procedures (i), (ii), and (iii) were repeated one hundred times for each sector. Figure 2 shows the procedures for calculating the permutation importance to the demographic sector as an example. We compared the difference in accuracy in each sector using the Mann-Whitney U test.

All analyses were performed using Python Language, version 3.8.2 (Python Software Foundation at http://www.python.org). Statistical significance was evaluated by 95%CI as indicated by a p-value of <0.05.

## Results

Table 1 shows the characteristics of the patients in the training and test datasets.

**Table 1:** Patient characteristics

Parameter	Training dataset 122 patients, 1,570 images	Test dataset 30 patients, 484 images
Mean age (95%CI), years	61.4 (37.5–85.5)	62.1 (39.3–85.0)
Sex, n (%)		
Men	86 (70.5)	27 (90.0)
Women	36 (29.5)	3 (10.0)
Pathologic diagnosis^a^, n (%)		
Glioblastoma, IDH-wildtype	118 (96.7)	30 (100.0)
Astrocytoma, IDH-mutant	4 (3.3)	0 (0.0)
WHO grade^a^, n (%)		
4	122 (100.0)	30 (100.0)
MGMT promoter methylation, n (%)		
Methylated	88 (72.1)	20 (66.7)
Unmethylated	34 (28.9)	10 (33.3)
Images per patient, n (95%CI)	12.86 (11.4–14.3)	16.1 (13.4–18.9)

^a^World Health Organization Classification of Central Nervous System Tumors, 5th edition

Abbreviations: 95% CI 95% confidence interval, MGMT O6-methylguanine-DNA methyl transferase, WHO World Health Organization, NA not applicable

### Image-based analysis

**Fig. 3:**
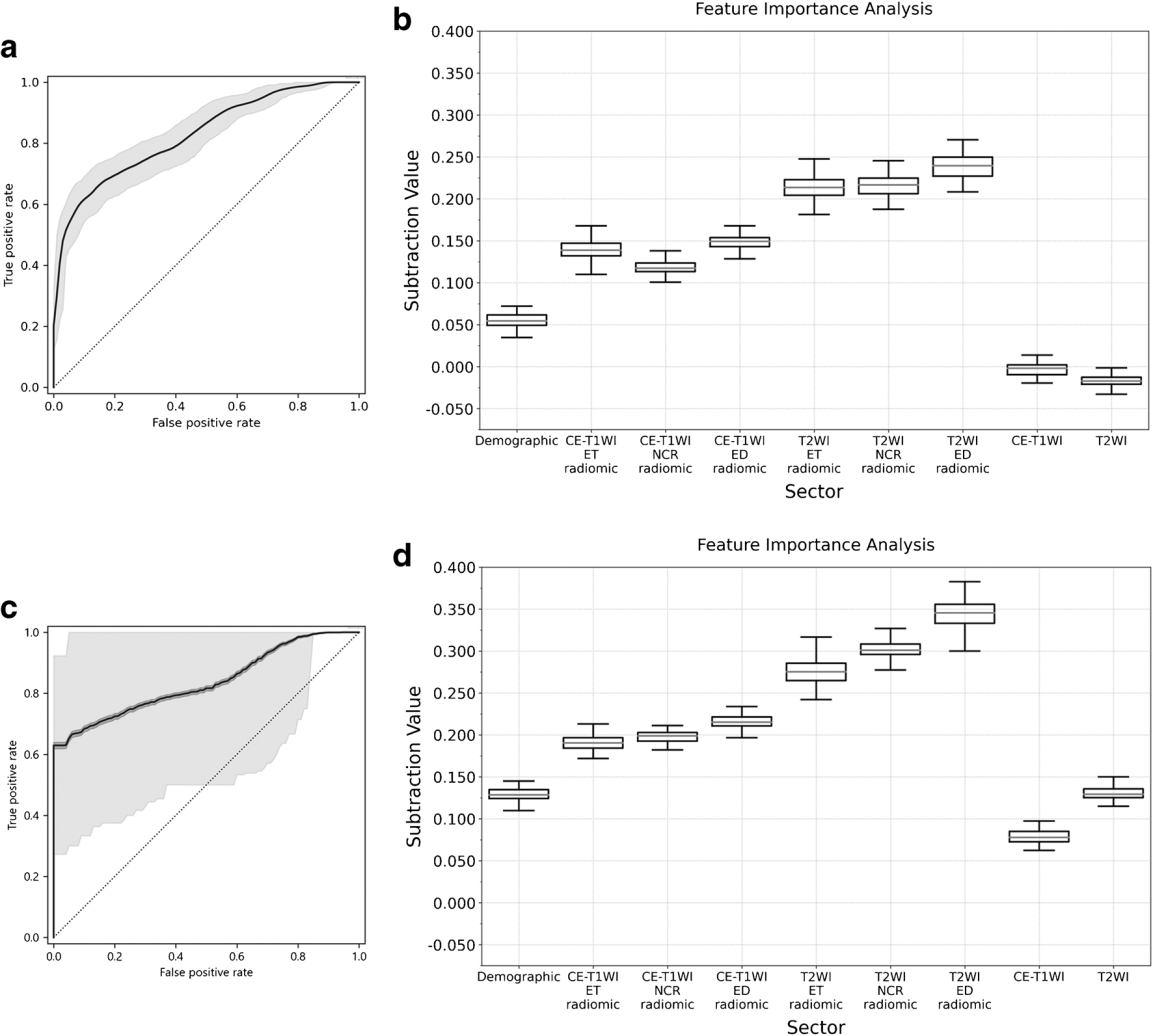
The receiver operating characteristic curve and results of permutation importance analysis for the test dataset. The curve of receiver-operating characteristics (ROC) of image-based analysis and patient-based analysis of the test dataset is shown in (**a**) and (**c**), respectively. The gray zone in each figure represents the 95% confidence interval (95%CI). The box plots of the difference between the original accuracy and accuracy calculated using the permutated dataset to respective sectors for image-base analysis and patient-base analysis are shown in (**b**) and (**d**), respectively. The horizontal line of the upper whisker, the upper horizontal line of the rectangle, the gray line, the lower horizontal line of the rectangle, and the horizontal line of the lower whisker represent the maximum, third quartile, median, first quartile, and minimum, respectively. Abbreviations: CE-T1WI: contrast-enhanced T1-weighted image; T2WI: T2-weighted image; ET: enhancing tumor; NCR: necrotic tumor core; ED: peritumoral edematous/infiltrated tissue

Accuracy, sensitivity, specificity, PPV, NPV, F-score, AUC-ROC, logarithmic loss, and Cohen’s κ coefficient of the total model output for the test dataset were 0.764 (95%CI: 0.743–0.782), 0.612 (95%CI: 0.579–0.641), 0.917 (95%CI: 0.892–0.930), 0.881 (95%CI: 0.846–0.899), 0.703 (95%CI: 0.674–0.726), 0.722 (95%CI: 0.682–0.762), 0.828 (95%CI: 0.792–0.862), 1.34 (95%CI: 1.34–1.35), and 0.529 (95%CI: 0.510–0.548), respectively. Figure 3a shows ROC of the total model output. Table 2a shows the statistics for each sector. Table 3a shows the performance of CNN models and the results of McNemar and DeLong tests. The CE-T1WI sectors had statistically different contingency tables compared with AlexNet (*p*<0.0001), GoogleNet (*p*=0.027), VGG16 (*p*<0.0001), and ResNet (*p*<0.0001). The T2WI sectors had statistically different contingency tables compared with AlexNet (*p*<0.0001), GoogleNet (*p*=0.031), VGG16 (*p*<0.0001), and ResNet (*p*<0.0001). vViT had higher accuracy compared with AlexNet, GoogleNet, VGG16, and ResNet for both CE-T1WI and T2WI. The AUC-ROC of CE-T1WI and T2WI sectors were higher than that of AlexNet, GoogleNet, VGG16, and ResNet (*p*<0.0001 for all). Sectors were ranked based on the permutation importance in descending order as T2WI ED radiomic (permutation importance = 0.239, 95%CI: 0.237–0.240), T2WI NCR radiomic (0.216, 95%CI: 0.214–0.217), T2WI ET radiomic (0.213, 95%CI: 0.211–0.214), CE-T1WI ED radiomic (0.148, 95%CI: 0.147–0.150), CE-T1WI ET radiomic (0.139, 95%CI: 0.137–0.140), CE-T1WI NCR radiomic (0.118, 95%CI: 0.116–0.120), demographic (0.0550, 95%CI: 0.0533–0.0566), T1WI (−0.0032, 95%CI: −0.0049 to −0.0015), and T2WI (−0.0169, 95%CI: −0.0186 to −0.0152) sectors. Figure 3b shows the results of the permutation feature importance of each sector for the test dataset. Table 4 shows the results of the Mann-Whitney U test for each combination of sectors, wherein the cells colored by dark gray in the upper triangle area present the combination of sectors which achieved a *p*-value of <0.0001 with the Mann-Whitney U test.

**Table 2:** Statistical data for each sector

Statistic (95%CI)	Total model output	Class token sector	Demographic sector	CE-T1WI ET radiomic sector	CE-T1WI NCR radiomic sector	CE-T1WI ED radiomic sector	T2WI ET radiomic sector	T2WI NCR radiomic sector	T2WI ED radiomic sector	CE-T1WI sector	T2WI sector
(a) Image-based analysis for the test dataset											
Accuracy	0.764 (0.743–0.782)	0.736 (0.714–0.754)	0.758 (0.737–0.776)	0.767 (0.745–0.784)	0.762 (0.741–0.780)	0.767 (0.745–0.784)	0.760 (0.739–0.778)	0.762 (0.741–0.780)	0.758 (0.737–0.776)	0.758 (0.737–0.776)	0.731 (0.710–0.750)
Sensitivity	0.612 (0.579–0.641)	0.566 (0.533–0.597)	0.607 (0.574–0.637)	0.603 (0.570–0.633)	0.616 (0.583–0.645)	0.607 (0.574–0.637)	0.624 (0.591–0.653)	0.616 (0.583–0.645)	0.607 (0.574–0.637)	0.620 (0.587–0.649)	0.492 (0.460–0.524)
Specificity	0.917 (0.892–0.930)	0.905 (0.879–0.918)	0.909 (0.883–0.922)	0.930 (0.905–0.941)	0.909 (0.883–0.922)	0.926 (0.901–0.937)	0.897 (0.870–0.911)	0.909 (0.883–0.922)	0.909 (0.883–0.922)	0.897 (0.870–0.911)	0.971 (0.951–0.977)
PPV	0.881 (0.846–0.899)	0.856 (0.819–0.877)	0.870 (0.834–0.889)	0.896 (0.861–0.912)	0.871 (0.836–0.890)	0.891 (0.856–0.908)	0.858 (0.823–0.878)	0.871 (0.836–0.890)	0.870 (0.834–0.889)	0.857 (0.822–0.877)	0.944 (0.907–0.956)
NPV	0.703 (0.674–0.726)	0.676 (0.648–0.700)	0.698 (0.670–0.722)	0.701 (0.673–0.724)	0.703 (0.675–0.726)	0.702 (0.674–0.725)	0.705 (0.676–0.728)	0.703 (0.675–0.726)	0.698 (0.670–0.722)	0.702 (0.674–0.726)	0.656 (0.630–0.680)
F-score	0.722 (0.682–0.762)	0.682 (0.640–0.723)	0.715 (0.675–0.756)	0.721 (0.681–0.761)	0.722 (0.682–0.761)	0.722 (0.682–0.762)	0.722 (0.683–0.762)	0.722 (0.682–0.761)	0.715 (0.675–0.756)	0.719 (0.679–0.759)	0.647 (0.604–0.689)
AUC-ROC	0.828 (0.792–0.862)	0.797 (0.758–0.835)	0.787 (0.747–0.829)	0.783 (0.743–0.825)	0.793 (0.753–0.835)	0.790 (0.749–0.832)	0.785 (0.744–0.828)	0.803 (0.763–0.842)	0.800 (0.760–0.840)	0.830 (0.794–0.863)	0.813 (0.777–0.852)
Logarithmic loss	1.34 (1.34–1.35)	2.07 (2.06–2.07)	2.39 (2.39–2.39)	2.62 (2.61–2.62)	2.53 (2.52–2.53)	2.50 (2.50–2.50)	2.49 (2.49–2.50)	2.50 (2.50–2.50)	2.58 (2.57–2.58)	0.955 (0.952–0.958)	1.59 (1.59–1.60)
Cohen’s Kappa score	0.529 (0.510–0.548)	0.471 (0.451–0.491)	0.517 (0.497–0.536)	0.533 (0.514–0.552)	0.525 (0.506–0.544)	0.533 (0.514–0.552)	0.521 (0.502–0.540)	0.525 (0.506–0.544)	0.517 (0.497–0.536)	0.517 (0.497–0.536)	0.463 (0.443–0.483)
(b) Patient-based analysis for the test dataset											
Accuracy	0.833 (0.714–0.877)	0.833 (0.714–0.877)	0.833 (0.714–0.877)	0.833 (0.714–0.877)	0.833 (0.714–0.877)	0.833 (0.714–0.877)	0.833 (0.714–0.877)	0.833 (0.714–0.877)	0.833 (0.714–0.877)	0.833 (0.714–0.877)	0.867 (0.747–0.903)
Sensitivity	0.600 (0.395–0.749)	0.600 (0.395–0.749)	0.600 (0.395–0.749)	0.600 (0.395–0.749)	0.600 (0.395–0.749)	0.600 (0.395–0.749)	0.600 (0.395–0.749)	0.600 (0.395–0.749)	0.600 (0.395–0.749)	0.600 (0.395–0.749)	0.600 (0.395–0.749)
Specificity	0.950 (0.788–0.967)	0.950 (0.788–0.967)	0.950 (0.788–0.967)	0.950 (0.788–0.967)	0.950 (0.788–0.967)	0.950 (0.788–0.967)	0.950 (0.788–0.967)	0.950 (0.788–0.967)	0.950 (0.788–0.967)	0.950 (0.788–0.967)	1.00 (0.839–1.000)
PPV	0.857 (0.535–0.927)	0.857 (0.535–0.927)	0.857 (0.535–0.927)	0.857 (0.535–0.927)	0.857 (0.535–0.927)	0.857 (0.535–0.927)	0.857 (0.535–0.927)	0.857 (0.535–0.927)	0.857 (0.535–0.927)	0.857 (0.535–0.927)	1.00 (0.610–1.000)
NPV	0.826 (0.682–0.877)	0.826 (0.682–0.877)	0.826 (0.682–0.877)	0.826 (0.682–0.877)	0.826 (0.682–0.877)	0.82E-T1TW (0.682–0.877)	0.826 (0.682–0.877)	0.826 (0.682–0.877)	0.826 (0.682–0.877)	0.826 (0.682–0.877)	0.833 (0.693–0.882)
F-score	0.706 (0.543–0.869)	0.706 (0.543–0.869)	0.706 (0.543–0.869)	0.706 (0.543–0.869)	0.706 (0.543–0.869)	0.706 (0.543–0.869)	0.706 (0.543–0.869)	0.706 (0.543–0.869)	0.706 (0.543–0.869)	0.706 (0.543–0.869)	0.750 (0.595–0.905)
AUC-ROC	0.840 (0.650–0.995)	0.825 (0.636–0.992)	0.820 (0.619–0.983)	0.810 (0.591–0.995)	0.825 (0.620–0.995)	0.830 (0.620–1.000)	0.845 (0.659–1.000)	0.840 (0.656–0.990)	0.815 (0.609–0.985)	0.840 (0.656–0.995)	0.835 (0.652–0.990)
Logarithmic loss	0.613 (0.561–0.665)	0.807 (0.750–0.863)	0.888 (0.833–0.943)	1.05 (0.990–1.11)	0.977 (0.920–1.03)	1.01 (0.954–1.07)	0.889 (0.835–0.944)	0.925 (0.868–0.981)	1.02 (0.959–1.08)	0.474 (0.418–0.530)	0.741 (0.683–0.798)
Cohen’s Kappa score	0.595 (0.540–0.649)	0.595 (0.540–0.649)	0.595 (0.540–0.649)	0.595 (0.540–0.649)	0.595 (0.540–0.649)	0.595 (0.540–0.649)	0.595 (0.540–0.649)	0.595 (0.540–0.649)	0.595 (0.540–0.649)	0.595 (0.540–0.649)	0.667 (0.618–0.715)

Abbreviations: 95%CI 95% confidence interval, PPV positive predictive value, NPV negative predictive value, AUC-ROC area under the curve of receiver-operating characteristics, CE-T1WI contrast-enhanced T1WI-weighted image, T2WI T2WI-weighted image, ET enhancing tumor, NCR necrotic tumor core, ED peritumoral edematous/infiltrated tissue

**Table 3:** Comparison of performance between vViT and CNN models

Statistics (95%CI)	CE-T1WI					T2WI				
	vViT	AlexNet	GoogleNet	VGG16	ResNet	vViT	AlexNet	GoogleNet	VGG16	ResNet
(a) Image-based analysis										
Accuracy	0.758 (0.737–0.776)	0.504 (0.482–0.527)	0.494 (0.471–0.516)	0.467 (0.445–0.490)	0.519 (0.496–0.541)	0.833 (0.714–0.877)	0.531 (0.508–0.553)	0.506 (0.484–0.529)	0.496 (0.473–0.518)	0.529 (0.506–0.551)
Sensitivity	0.620 (0.587–0.649)	0.091 (0.078–0.117)	0.430 (0.399–0.463)	0.636 (0.603–0.665)	0.698 (0.666–0.725)	0.600 (0.395–0.749)	0.459 (0.427–0.491)	0.331 (0.303–0.363)	0.888 (0.861–0.903)	0.698 (0.666–0.725)
Specificity	0.897 (0.870–0.911)	0.917 (0.892–0.930)	0.558 (0.525–0.589)	0.298 (0.271–0.330)	0.339 (0.311–0.371)	0.950 (0.788–0.967)	0.603 (0.570–0.633)	0.682 (0.649–0.709)	0.103 (0.089–0.130)	0.360 (0.331–0.392)
PPV	0.857 (0.822–0.877)	0.524 (0.441–0.603)	0.493 (0.459–0.527)	0.475 (0.448–0.503)	0.514 (0.486–0.541)	0.857 (0.535–0.927)	0.536 (0.501–0.570)	0.510 (0.469–0.549)	0.498 (0.474–0.521)	0.522 (0.494–0.549)
NPV	0.702 (0.674–0.726)	0.502 (0.479–0.526)	0.495 (0.465–0.525)	0.450 (0.412–0.491)	0.529 (0.488–0.569)	0.826 (0.682–0.877)	0.527 (0.497–0.556)	0.505 (0.477–0.532)	0.481 (0.410–0.554)	0.544 (0.503–0.582)
F-score	0.719 (0.679–0.759)	0.155 (0.123–0.187)	0.459 (0.415–0.504)	0.544 (0.500–0.589)	0.592 (0.548–0.636)	0.706 (0.543–0.869)	0.494 (0.450–0.539)	0.401 (0.357–0.445)	0.638 (0.595–0.681)	0.597 (0.553–0.641)
*p*-value	-	<0.0001	0.027	<0.0001	<0.0001	-	<0.0001	0.031	<0.0001	<0.0001
AUC-ROC	0.830 (0.794–0.863)	0.485 (0.437-0.534)	0.483 (0.434-0.532)	0.504 (0.455-0.553)	0.520 (0.471-0.569)	0.813 (0.777–0.852)	0.502 (0.453-0.551)	0.535 (0.486-0.584)	0.547 (0.498-0.596)	0.512 (0.463-0.562)
*p*-value	-	<0.0001	<0.0001	<0.0001	<0.0001	-	<0.0001	<0.0001	<0.0001	<0.0001
(b) Patient-based analysis										
Accuracy	0.833 (0.714–0.877)	0.667 (0.554–0.742)	0.467 (0.373–0.568)	0.367 (0.287–0.477)	0.400 (0.315–0.508)	0.867 (0.747–0.903)	0.633 (0.523–0.713)	0.600 (0.492–0.685)	0.333 (0.258–0.446)	0.467 (0.373–0.568)
Sensitivity	0.600 (0.395–0.749)	0.000 (0.000–0.278)	0.000 (0.000–0.278)	1.000 (0.722–1.000)	1.000 (0.722–1.000)	0.600 (0.395–0.749)	0.300 (0.183–0.528)	0.000 (0.000–0.278)	1.000 (0.722–1.000)	1.000 (0.722–1.000)
Specificity	0.950 (0.788–0.967)	1.000 (0.839–1.000)	0.700 (0.551–0.784)	0.050 (0.033–0.212)	0.100 (0.067–0.262)	1.00 (0.839–1.000)	0.800 (0.643–0.861)	0.900 (0.738–0.933)	0.000 (0.000–0.161)	0.200 (0.139–0.357)
PPV	0.857 (0.535–0.927)	-	0.000 (0.000–0.390)	0.345 (0.267–0.459)	0.357 (0.276–0.473)	1.00 (0.610–1.000)	0.429 (0.241–0.667)	0.000 (0.000–0.658)	0.333 (0.258–0.446)	0.385 (0.296–0.503)
NPV	0.826 (0.682–0.877)	0.667 (0.554–0.742)	0.583 (0.462–0.681)	1.000 (0.207–1.000)	1.000 (0.342–1.000)	0.833 (0.693–0.882)	0.696 (0.560–0.775)	0.643 (0.527–0.724)	-	1.000 (0.510–1.000)
* F*-score	0.706 (0.543–0.869)	-	-	0.513 (0.334–0.692)	0.526 (0.348–0.705)	0.750 (0.595–0.905)	0.353 (0.182–0.524)	-	0.500 (0.321–0.679)	0.556 (0.378–0.733)
*p*-value	-	0.016	0.21	<0.0001	<0.0001	-	1.0	1.0	<0.0001	<0.0001
AUC-ROC	0.840 (0.656–0.995)	0.490 (0.238-0.742)	0.530 (0.319-0.741)	0.635 (0.424-0.846)	0.615 (0.399-0.831)	0.835 (0.652–0.990)	0.535 (0.321-0.749)	0.525 (0.317-0.733)	0.67 (0.476-0.864)	0.525 (0.313-0.737)
*p*-value	-	0.092	0.048	0.367	0.26	-	0.032	0.025	0.28	0.045

Abbreviations: CE-T1WI contrast-enhanced T1WI-weighted image, T2WI T2WI-weighted image, 95%CI 95% confidence interval, vViT variable Vision Transformer, PPV positive predictive value, NPV negative predictive value, AUC-ROC area under the curve of receiver-operating characteristic, VGG Visual Geometry Group

Statistics or p-value cannot be calculated was represented as a hyphen (-)

**Table 4:**
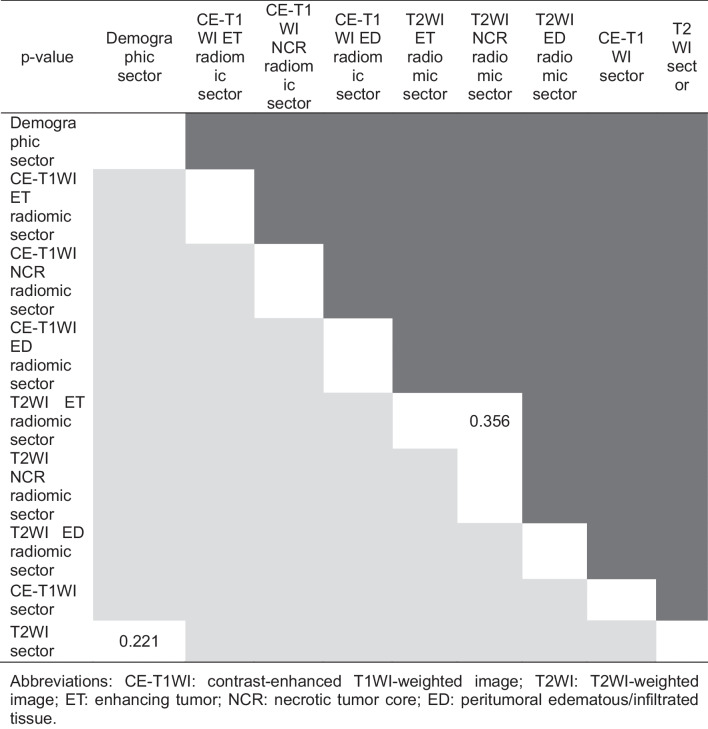
The results of the Mann-Whitney U test for the difference between original accuracy and accuracy were calculated using a permutated dataset. The cells colored by dark and light gray areas present the combination of sectors which gave *p*< 0.0001 by the Mann–Whitney U test for image base and patient base analyses, respectively: image-based analysis  and patient-based analysis  .

Abbreviations: CE-T1WI contrast-enhanced T1WI-weighted image, T2WI T2WI-weighted image, ET enhancing tumor, NCR necrotic tumor core, ED peritumoral edematous/infiltrated tissue

### Patient-based analysis

Accuracy, sensitivity, specificity, PPV, NPV, F-score, AUC-ROC, logarithmic loss, and Cohen’s κ coefficient of the total model output for the test dataset were 0.833 (95%CI: 0.714–0.877), 0.600 (95%CI: 0.395–0.749), 0.950 (95%CI: 0.788–0.967), 0.857 (95%CI: 0.535–0.927), 0.826 (95%CI: 0.682–0.877), 0.706 (95%CI: 0.543–0.869), 0.840 (95%CI: 0.650–0.995), 0.613 (95%CI: 0.561–0.665), and 0.595 (95%CI: 0.540–0.649), respectively. Figure 3c shows ROC of the total model output. Table 2b shows the statistics of the total model output and each sector. Table 3b shows the performance of CNN models and the results of McNemar and DeLong tests. The CE-T1WI sector of vViT had statistically different contingency table compared with AlexNet (*p*=0.016), VGG16 (*p*<0.0001), and ResNet (*p*<0.0001). The accuracy of the CE-T1 sector was higher than that of AlexNet, VGG16, and ResNet. The AUC-ROC of the CE-T1WI sector was higher than that of GoogleNet (*p*=0.048). The T2WI sector had statistically different contingency table compared with VGG16 (*p*<0.0001) and ResNet (*p*<0.0001). The accuracy of T2WI sector was higher than that of VGG16. The AUC-ROC of the CE-T1WI sector was statistically higher than that of GoogleNet (*p*=0.0048). The AUC-ROC of the T2WI sector was statistically higher than that of AlexNet (*p*=0.032), GoogleNet (*p*=0.025), and ResNet (*p*=0.045). Sectors were ranked based on the importance in descending order as T2WI ED radiomic (permutation importance = 0.345, 95%CI: 0.343–0.346), T2WI NCR radiomic (0.302, 95%CI: 0.301–0.304), T2WI ET radiomic (0.276, 95%CI: 0.274–0.277), CE-T1WI ED radiomic (0.216, 95%CI: 0.214–0.217), CE-T1WI NCR radiomic (0.197, 95%CI: 0.196–0.199), CE-T1WI ET radiomic (0.191, 95%CI: 0.189–0.192), T2WI (0.131, 95%CI: 0.0772–0.0801), demographic (0.129, 95%CI: 0.128–0.131), and T1WI (0.0787, 95%CI: 0.0772–0.0801) sectors. Figure 3d shows the results of the permutation feature importance of each sector for the test dataset. Table 4 shows the results of the Mann-Whitney U test for each combination of sectors, wherein the cells colored by light gray in the lower triangle area represent the combination of sectors which achieved a *p*-value of <0.0001 by the Mann-Whitney U test.

## Discussion

The present study aimed to, first, examine the performance of vViT in predicting MGMT promoter methylation among adult patients with diffuse glioma, using demographics, radiomic features, and MRI itself and, second, identify the dominant factor among demographics, radiomic features, and MRI itself. To the best of our knowledge, this is the first study to apply vViT in predicting MGMT promoter methylation and to determine the dominant factor among demographics, radiomic features, and MRI itself. The vViT in the patient-base analysis demonstrated an accuracy of 0.833 and AUC-ROC of 0.840 for the test dataset. Robinet et al. applied EmbraceNet to predict MGMT promoter methylation using T1WI and fluid-attenuated inversion recovery (FLAIR) images [37]. Robinet et al. revealed slightly higher performance than random and concluded the poor performance of current deep learning methods in determining MGMT promoter methylation from MRI. Moreover, the BraTS21 competition, which was hosted by the Radiological Society of North America (RSNA) and the Medical Image Computing and Computer-Assisted Interventions, achieved first place with an accuracy of 0.67 and AUC-ROC of 0.62 [21]. The dataset we used was partly overlapped with the dataset used in this competition. Regarding this point, vViT achieved higher performance than previously reported. In this study, there was no CNN model that achieved statistically higher accuracy and AUC-ROC compared with vViT. On the other hand, Yogananda et al. and Korfiatis et al. reported 0.947 and 0.949 of accuracies using CNN in predicting MGMT promoter methylation, respectively [17; 38]. The inconsistency may be caused by differences in study population characteristics and the classification method. Hence, the reproducibility of each method should be continuously confirmed. In a recent study, Xu et al. achieved 0.952 of accuracy by ViT using CE-T1WI and T2WI [38]. Although the performance was overestimated due to the imbalance of the dataset, the result indicates that the multimodal approach by Transformer is promising in predicting MGMT status. In the present study, we revealed that the multi-modality analysis reached higher performance than CNNs in predicting MGMT promoter methylation although vViT could not achieve state-of-the-art performance. The development of effective multimodal fusion approaches is becoming increasingly important to capture features of complex diseases [39]. Predicting MGMT promoter methylation among adult patients with diffuse glioma is not an exception.

The dominance of radiomic features or MRI itself in predicting MGMT promoter methylation remains controversial. The radiomic features tend to be analyzed using a machine learning algorithm [12, 14–16], while MRI tends to be analyzed using CNN [17–21]. The present study compared the permutation importance of radiomic features and MRI by vViT. By evaluating the importance of each sector, our vViT overcomes a difficulty of CNN: convoluting the input values makes it difficult to estimate the importance of input factors in making predictions although conventional CNN can handle multiple factors. We revealed that the radiomic features of ED had the highest importance in both image-based and patient-based analysis. A previous study indicated that the heterogeneity of edema region may have key information on MGMT promoter methylation [2]. As Yang et al. mentioned in this study, radiomics may be a promising technique to evaluate the heterogeneity in the edema region. Other studies insisted that the edema region represents the aggressive degree of glioma [40, 41]. The result of the present study reconfirmed these previous studies.

The present study has some limitations. First, we used the retrospectively collected dataset in a single center. The generalizability of the present study should be validated using another dataset. If there are errors such as data duplication or misidentification, the errors may not be identified. Of course, there is another open dataset that collected data from patients with glioma [42]. However, predicting MGMT promoter methylation is a challenging task and there remains inconsistency in performance. This inconsistency can be explained by differences among datasets or methods of image preprocessing as well as the performance of the deep learning model. Another dataset collected under different criteria and imaging conditions may be inappropriate for validation [43]. It cannot be stated that vViT is applicable in a clinical setting based on the results of this study alone. A comparison of vViT with radiologists or diagnostic improvement by radiologists using vViT should be examined in a future study. Second, the patient-based analysis revealed inequality between the numbers of patients. The training and test datasets were constructed to have the same number of images to input as many as possible number of images into the vViT. By this equalization, the overestimation of performance was avoided. This development led to inequality and selection bias, with an overestimated performance of the patient-based analysis. However, the performance of image-based analysis was partly comparable with previously reported MGMT prediction. This effect may be limited. Third, the radiomic and image sectors demonstrated an imbalance. The vViT had six radiomic and two image sectors in predicting MGMT promoter methylation. The imbalance between the number of radiomic and image sectors in vViT may make findings of radiomic features dominant. Changing the implementation setting in split-sequence and linear projection may be a solution. When the parameters of vViT and the number of radiomics are changed, the performance can be improved. As far as we investigated, the best performance was obtained when 64 radiomic features were used. Fourth, the interpretability of each demographic and radiomic feature was insufficient. We were not able to determine the contribution of each radiomic feature included in the T2WI ED radiomic sector. This point leads to difficulty in clarifying how the edema region was evaluated by radiomics. However, we can speculate the dominant radiomic feature by the F-value shown in Appendix 3. In addition to this, the biological meaning of each feature can be checked by a document [31].

In conclusion, vViT can be a competent model for predicting MGMT promoter methylation among adult patients with diffuse glioma compared with conventional CNN models. The input factors can be ranked by combining vViT with permutation feature importance. The most dominant factor among demographics, radiomic features, and MRI in predicting MGMT promoter methylation was the radiomic features derived from the edema region in T2WI for both image- and patient-based analysis. The radiomic features derived from CE-T1WI and T2WI had statistically higher importance than CE-T1WI and T2WI itself in predicting MGMT promoter methylation. The present study demonstrates that radiomic features have higher permutation importance in predicting MGMT promoter methylation compared with MRI itself.

### Supplementary information

ESM 1 (DOCX 1286 kb)

## Abbreviations

95%CI: 95% confidence interval
AUC-ROC: area under the curve of receiver-operating characteristics
CE-T1WI: T1-weighted images
CNN: convolutional neural network
CNS: central nervous system
ED: peritumoral edematous/infiltrated tissue
ET: enhancing tumor
MGMT: O6-methylguanine-DNA methyl transferase
MRI: magnetic resonance imaging
NCR: necrotic tumor core
NPV: negative predictive value
PPV: positive predictive value
T2WI: T2-weighted images
UCSF-PDGM: University of California San Francisco preoperative diffuse glioma magnetic resonance imaging
WHO: World Health Organization
vViT: variable vision transformer
ViT: Vision transformer
VGG16: visual geometry group 16

## References

[1] Ostrom QT, Gittleman H, Fulop J (2015). CBTRUS Statistical Report: primary brain and central nervous system tumors diagnosed in the United States in 2008-2012. Neuro-Oncol.

[2] Yang Y, Han Y, Zhao S (2022). Spatial heterogeneity of edema region uncovers survival-relevant habitat of Glioblastoma. Eur J Radiol.

[3] Louis DN, Perry A, Wesseling P (2021). The 2021 WHO classification of tumors of the central nervous system: a summary. Neuro-Oncol.

[4] The 2021 WHO classification of tumors of the central nervous system (5th ed.)

[5] Yan H, Parsons DW, Jin G (2009). IDH1 and IDH2 mutations in gliomas. N Engl J Med.

[6] Eckel-Passow JE, Lachance DH, Molinaro AM (2015). Glioma groups based on 1p/19q, IDH, and TERT promoter mutations in tumors. N Engl J Med.

[7] Esteller M, Herman JG (2004). Generating mutations but providing chemosensitivity: the role of O6-methylguanine DNA methyltransferase in human cancer. Oncogene.

[8] Hegi ME, Diserens AC, Gorlia T (2005). MGMT gene silencing and benefit from temozolomide in glioblastoma. N Engl J Med.

[9] Wick W, Platten M, Meisner C (2012). Temozolomide chemotherapy alone versus radiotherapy alone for malignant astrocytoma in the elderly: the NOA-08 randomised, phase 3 trial. Lancet Oncol.

[10] Weller M, Van Den Bent M, Preusser M (2021). EANO guidelines on the diagnosis and treatment of diffuse gliomas of adulthood. Nat Rev Clin Oncol.

[11] Vagvala S, Guenette JP, Jaimes C, Huang RY (2022) Imaging diagnosis and treatment selection for brain tumors in the era of molecular therapeutics. Cancer Imaging 22:19 10.1186/s40644-022-00455-5 PMC9014574 35436952

[12] Drabycz S, Roldan G, de Robles P (2010). An analysis of image texture, tumor location, and MGMT promoter methylation in glioblastoma using magnetic resonance imaging. Neuroimage.

[13] Kickingereder P, Bonekamp D, Nowosielski M (2016). Radiogenomics of Glioblastoma: machine learning-based classification of molecular characteristics by using multiparametric and multiregional mr imaging features. Radiology.

[14] Korfiatis P, Erickson B (2019). Deep learning can see the unseeable: predicting molecular markers from MRI of brain gliomas. Clin Radiol.

[15] Le NQK, Do DT, Chiu FY, Yapp EKY, Yeh HY, Chen CY (2020) XGBoost improves classification of MGMT promoter methylation status in IDH1 wildtype glioblastoma. J Pers Med 10(3):128 10.3390/jpm10030128 PMC7563334 32942564

[16] Do DT, Yang MR, Lam LHT, Le NQK, Wu YW (2022). Improving MGMT methylation status prediction of glioblastoma through optimizing radiomics features using genetic algorithm-based machine learning approach. Sci Rep.

[17] Korfiatis P, Kline TL, Lachance DH, Parney IF, Buckner JC, Erickson BJ (2017). Residual deep convolutional neural network predicts mgmt methylation status. J Digit Imaging.

[18] Chang P, Grinband J, Weinberg BD (2018). Deep-learning convolutional neural networks accurately classify genetic mutations in gliomas. Am J Neuroradiol.

[19] Han L, Kamdar MR (2018) MRI to MGMT: predicting methylation status in glioblastoma patients using convolutional recurrent neural networks. 10.1142/9789813235533_0031:331-342 PMC5728677 29218894

[20] Yogananda CGB, Shah BR, Nalawade SS (2021). MRI-based deep-learning method for determining glioma mgmt promoter methylation status. Am J Neuroradiol.

[21] Kim BH, Lee H, Choi KS et al (2022) Validation of MRI-based models to predict MGMT promoter methylation in gliomas: BraTS 2021 radiogenomics challenge. Cancers 14(19):4827 10.3390/cancers14194827 PMC9562637 36230750

[22] Usuzaki T (2022) Splitting expands the application range of vision transformer -- variable vision transformer (vViT). arXiv.2211.03992

[23] Acosta JN, Falcone GJ, Rajpurkar P, Topol EJ (2022). Multimodal biomedical AI. Nat Med.

[24] Rudin C (2019). Stop explaining black box machine learning models for high stakes decisions and use interpretable models instead. Nat Mach Intell.

[25] Vaswani A, Shazeer N, Parmar N et al (2017) Attention is all you need. 10.48550/ARXIV.1706.03762

[26] Dosovitskiy A, Beyer L, Kolesnikov A et al (2020) An image is worth 16x16 words: transformers for image recognition at scale. 10.48550/ARXIV.2010.11929

[27] Usuzaki T, Takahashi K, Inamori R (2024). Grading diffuse glioma based on 2021 WHO grade using self-attention-base deep learning architecture: variable Vision Transformer (vViT). Biomed Signal Process Control.

[28] Calabrese E, Villanueva-Meyer JE, Rudie JD (2022). The University of California San Francisco preoperative diffuse glioma MRI dataset. Radiol: Artif Intell.

[29] Clark K, Vendt B, Smith K (2013). The cancer imaging archive (TCIA): maintaining and operating a public information repository. J Digit Imaging.

[30] Bakas S, Reyes M, Jakab A et al (2018) Identifying the best machine learning algorithms for brain tumor segmentation, progression assessment, and overall survival prediction in the brats challenge. arXiv:1811.02629

[31] van Griethuysen JJM, Fedorov A, Parmar C (2017). Computational radiomics system to decode the radiographic phenotype. Cancer Res.

[32] Krizhevsky A, Sutskever I, Hinton GE, Pereira F, Burges CJ, Bottou L, Weinberger KQ (2012). ImageNet classification with deep convolutional neural networks. Advances in neural information processing systems.

[33] Szegedy C, Liu W, Jia Y et al (2014) Going deeper with convolutions. arXiv:1409.4842

[34] Simonyan K, Zisserman A (2015) Very deep convolutional networks for large-scale image recognition. arXiv:1409.1556

[35] He K, Zhang X, Ren S, Sun J (2015) Deep residual learning for image recognition. arXiv:1512.03385

[36] Usuzaki T, Ishikuro M, Murakami K (2020). How can we evaluate whether an association is truly inter-generational?. J Hypertens.

[37] Robinet L, Siegfried A, Roques M, Berjaoui A, Cohen-Jonathan Moyal E (2023) MRI-based deep learning tools for mgmt promoter methylation detection: a thorough evaluation. Cancers 15(8):2253 10.3390/cancers15082253 PMC10137327 37190181

[38] Xu Q, Xu QQ, Shi N, Dong LN, Zhu H, Xu K (2022). A multitask classification framework based on vision transformer for predicting molecular expressions of glioma. Eur J Radiol.

[39] Steyaert S, Pizurica M, Nagaraj D (2023). Multimodal data fusion for cancer biomarker discovery with deep learning. Nat Mach Intell.

[40] Lemee JM, Clavreul A, Menei P (2015). Intratumoral heterogeneity in glioblastoma: don't forget the peritumoral brain zone. Neuro-Oncol.

[41] Bakas S, Akbari H, Pisapia J (2017). In vivo detection of EGFRvIII in glioblastoma via perfusion magnetic resonance imaging signature consistent with deep peritumoral infiltration: the phi-index. Clin Cancer Res.

[42] Bakas S, Sako C, Akbari H (2022). The University of Pennsylvania glioblastoma (UPenn-GBM) cohort: advanced MRI, clinical, genomics, & radiomics. Sci Data.

[43] Shamir GI, Lin D (2022) Real world large scale recommendation systems reproducibility and smooth activations. arXiv:2202.06499

