# Supplementary material for: Identifying key factors for predicting O6-Methylguanine-DNA methyltransferase status in adult patients with diffuse glioma: a multimodal analysis of demographics, radiomics, and MRI by variable Vision Transformer
Source: Neuroradiology. 2024 Mar 12;66(5):761–73. doi: 10.1007/s00234-024-03329-8 (PMC11031474; doi:10.1007/s00234-024-03329-8)
Supplement: Supplementary file 1 — (DOCX 1286 kb) [file 234_2024_3329_MOESM1_ESM.docx]

**Appendix 1**. The list of 105 radiomic features and explanations of each category [1, 2]

***First-order features***

Explanation:

First order texture features describe as the simplest information contained in the imagery, which are calculated from the gray-level histogram of an image (i.e., information about the distribution and frequency of pixels with specific intensity within a ROI).

List of First-order features (the number of features, 20):

The number of pixels, energy, total energy, entropy, minimum, 10 percentile, 90 percentile, maximum, mean, median, range, interquartile range, mean absolute deviation, robust mean absolute deviation, root mean squared, standard deviation, skewness, kurtosis, variance, and uniformity.

***Shape2D features***

Explanation:

Shape2D texture features describe as the two-dimensional size and shape of the ROI, which are independent from the gray level intensity distribution in the ROI and are therefore only calculated on the non-derived image and mask.

List of Shapw2D features (10):

Mesh surface, pixel surface, perimeter, perimeter surface ratio, sphericity, spherical disproportion, maximum diameter, major axis length, minor axis length, and elongation.

***Gray-Level Co-occurrence Matrix (GLCM) features***

Explanation:

Gray-Level Co-occurence Matrix (GLCM) texture features describe second-order statistical information of the image, which is defined as a matrix to indicate statistical interrelationships between voxels with similar or dissimilar signal intensity values within an imaging plane.

List of GLCM features (24)

Autocorrelation, joint average, cluster prominence, cluster shade, cluster tendency, contrast, correlation, difference average, difference entropy, difference variance, joint energy, joint entropy, informational measure of correlation 1, informational measure of correlation 2, inverse difference moment, maximal correlation coefficient, inverse difference moment normalized, inverse difference, inverse difference normalized, inverse variance, maximum probability, sum average, sum entropy, and sum squares.

***Gray-level Size Zone Matrix (GLSZM) features***

Explanation:

Gray-Level Size Zone Matrix (GLSZM) texture features describe regional high-order statistical information of the image, which is defined as a matrix to depict distribution of similar and dissimilar regions with intensity variations.

List of GLSZM features (16)

Small area emphasis, large area emphasis, gray-level nonuniformity, gray-level nonuniformity normalized, size zone nonuniformity, size zone nonuniformity normalized, zone percentage, gray-level variance, zone variance, zone entropy, low gray-level zone emphasis, high gray-level zone emphasis, small area low gray-level emphasis, small area high gray-level emphasis, large area low gray-level emphasis, and large area high gray-level emphasis.

***Gray-level Run Length Matrix (GLRLM) features***

Explanation:

Gray-Level Run-Length Matrix (GLRLM) texture features describe regional high-order statistical information of the image, which is defined as a matrix to express signal intensity and spatial interrelationship along a longitudinal run of neighboring voxels.

List of GLRLM features (16)

Short-run emphasis, long-run emphasis, gray-level nonuniformity, gray-level nonuniformity normalized, run length nonuniformity, run length nonuniformity normalized, run percentage, gray-level variance, run entropy, run variance, low gray-level run emphasis, high gray-level run emphasis, short-run low gray-level emphasis, short-run high gray-level emphasis, long run low gray-level emphasis, and long-run high gray-level emphasis.

***Neighboring Gray Tone Difference Matrix (NGTDM) features***

Explanation:

Neighbourhood Gray-Tone Difference Matrix (NGTDM) texture features describe high-order statistical information of the image, which is defined as a matrix to indicate intensity and spatial interrelationship between neighboring voxels in adjacent image planes.

List of NGTDM features (5)

Busyness, coarseness, complexity, contrast, and strength.

***Gray-level Dependence Matrix (GLDM) features***

Explanation:

Gray Level Dependence Matrix (GLDM) texture features describe high-order statistical information of the image, which is defined as a the number of connected voxels that are dependent on the center voxel.

List of GLDM features (14)

Small dependence emphasis, large dependence emphasis, gray-level nonuniformity, dependence nonuniformity, dependence nonuniformity normalized, gray-level variance, dependence variance, dependence entropy, low gray-level emphasis, high gray-level emphasis, small dependence low gray-level emphasis, small dependence high gray-level emphasis, large dependence low gray-level emphasis, and large dependence high gray-level emphasis.

**Appendix 2**

**Supplementary Figure 1**. The number of patients and images excluded by exclusion criteria and random selection. The figure shows exclusion and selection processes in predicting MGMT status for the UCSF-PDGM (training and test datasets). Abbreviations: CE-T1WI: contrast-enhanced T1-weighted image; T2WI: T2-weighted image; ET: enhancing tumor; NCR: necrotic tumor core; ED: peritumoral edematous/infiltrated tissue; MGMT: O6-methylguanine-DNA methyl transferase; UCSF-PDGM: University of California San Francisco Preoperative Diffuse Glioma MRI.


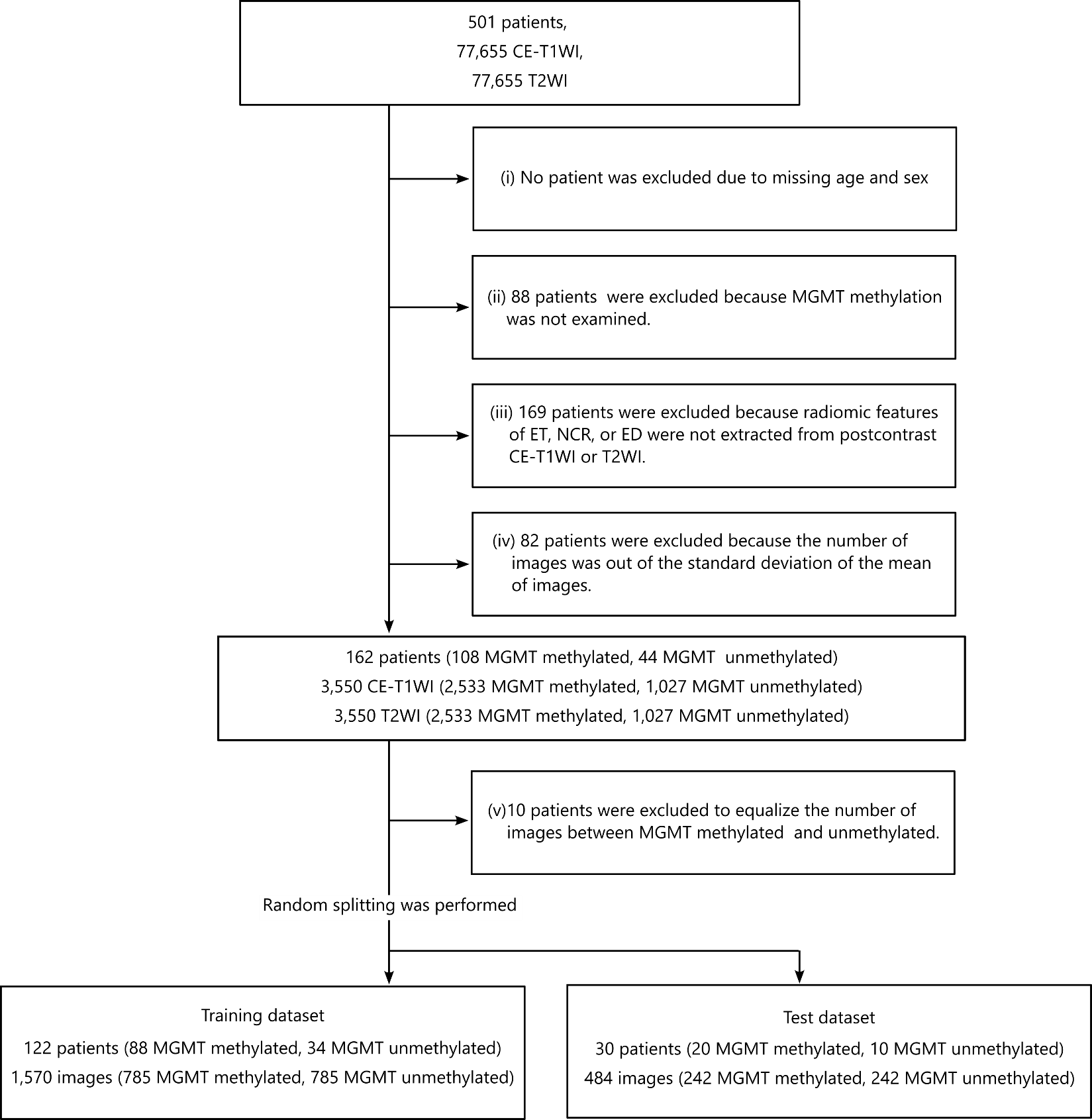


**Appendix 3.** The list of selected radiomic features, chi-square value, and *p*-value for MGMT status

**Supplementary Table 1.** Radiomic features selected from ET of contrast-enhanced T1-weighted image

| Area | Feature class | Feature | F-value | p-value |
| --- | --- | --- | --- | --- |
| ET  (contrast-enhanced  T1-weighted  image) | First Order | pixels T1enhance | 55.9 | p<0.001 |
|  |  | Entropy | 5.75 | 0.0166 |
|  |  | Minimum | 28.2 | p<0.001 |
|  |  | 10 Percentile | 22.2 | p<0.001 |
|  |  | 90 Percentile | 12.7 | p<0.001 |
|  |  | Maximum | 15.0 | p<0.001 |
|  |  | Mean | 16.3 | p<0.001 |
|  |  | Median | 14.5 | p<0.001 |
|  |  | Root Mean Squared | 15.5 | p<0.001 |
|  |  | Uniformity | 11.8 | p<0.001 |
|  | Shape 2D | Mesh Surface | 55.5 | p<0.001 |
|  |  | Pixel Surface | 55.9 | p<0.001 |
|  |  | Perimeter | 20.0 | p<0.001 |
|  |  | Perimeter Surface Ratio | 35.9 | p<0.001 |
|  |  | Maximum Diameter | 14.3 | p<0.001 |
|  |  | Major Axis Length | 12.5 | p<0.001 |
|  |  | Minor Axis Length | 10.7 | 0.00110 |
|  | GLCM | Contrast | 6.30 | 0.0122 |
|  |  | Correlation | 6.88 | 0.00879 |
|  |  | Difference Average | 11.7 | p<0.001 |
|  |  | Difference Entropy | 13.2 | p<0.001 |
|  |  | Difference Variance | 6.51 | 0.0108 |
|  |  | Joint Entropy | 11.3 | p<0.001 |
|  |  | Informational Measure of Correlation 1 | 46.4 | p<0.001 |
|  |  | Informational Measure of Correlation 2 | 16.0 | p<0.001 |
|  |  | Inverse Difference Moment | 23.0 | p<0.001 |
|  |  | Maximal Correlation Coefficient | 16.4 | p<0.001 |
|  |  | Inverse Difference | 25.0 | p<0.001 |
|  |  | Inverse Difference Normalized | 8.47 | 0.00366 |
|  |  | Inverse Variance | 23.5 | p<0.001 |
|  |  | Maximum Probability | 9.85 | 0.00173 |
|  | GLSZM | Large Area Emphasis | 8.08 | p<0.001 |
|  |  | Small Area Emphasis | 23.5 | 0.00452 |
|  |  | Gray Level Nonuniformity | 51.1 | p<0.001 |
|  |  | Gray Level Nonuniformity Normalized | 11.9 | p<0.001 |
|  |  | Size Zone Nonuniformity | 32.8 | p<0.001 |
|  |  | Size Zone Nonuniformity Normalized | 26.4 | p<0.001 |
|  |  | Zone Variance | 7.90 | p<0.001 |
|  |  | Zone Percentage | 24.7 | 0.00502 |
|  |  | Low Gray Level Zone Emphasis | 6.45 | 0.0112 |
|  |  | Large Area Low Gray Level Emphasis | 8.53 | 0.00354 |
|  | GLRLM | Long Run Emphasis | 13.4 | p<0.001 |
|  |  | Short Run Emphasis | 19.7 | p<0.001 |
|  |  | Gray Level Nonuniformity | 30.2 | p<0.001 |
|  |  | Gray Level Nonuniformity Normalized | 11.8 | p<0.001 |
|  |  | Run Length Nonuniformity | 44.9 | p<0.001 |
|  |  | Run Length Nonuniformity Normalized | 23.0 | p<0.001 |
|  |  | Run Percentage | 19.9 | p<0.001 |
|  |  | Run Variance | 12.5 | p<0.001 |
|  |  | Low Gray Level Run Emphasis | 8.35 | 0.00391 |
|  |  | Short Run Low Gray Level Emphasis | 6.33 | 0.0120 |
|  |  | Long Run Low Gray Level Emphasis | 12.5 | p<0.001 |
|  | NGTDM | Busyness | 9.47 | 0.00212 |
|  |  | Coarseness | 22.5 | p<0.001 |
|  |  | Contrast | 19.2 | p<0.001 |
|  |  | Strength | 6.81 | 0.00913 |
|  | GLDM | Small Dependence Emphasis | 27.1 | p<0.001 |
|  |  | Large Dependence Emphasis | 15.7 | p<0.001 |
|  |  | Gray Level Nonuniformity | 22.5 | p<0.001 |
|  |  | Dependence Nonuniformity | 30.9 | p<0.001 |
|  |  | Dependence Nonuniformity Normalized | 31.5 | p<0.001 |
|  |  | Dependence Variance | 22.7 | p<0.001 |
|  |  | Low Gray-level Emphasis | 10.9 | p<0.001 |
|  |  | Large Dependence Low Gray Level Emphasis | 12.7 | p<0.001 |

Abbreviations: ET: enhancing tumor; NCR: tumor core; ED: peritumoral edematous/infiltrated tissue; GLCM: gray-level co-occurrence matrix; GLSZM: gray-level size zone matrix; GLRLM: gray-level run-length matrix; NGTDM: neighboring gray-tone difference matrix; GLDM: gray-level dependence matrix.

**Supplementary Table 2.** Radiomic features selected from NCR of contrast-enhanced T1-weighted image

| Area | Feature class | Feature | F-value | p-value |
| --- | --- | --- | --- | --- |
| NCR  (contrast-enhanced  T1-weighted  image) | First Order | Energy | 2.89 | 0.0893 |
|  |  | Total Energy | 2.89 | 0.0893 |
|  |  | Entropy | 2.49 | 0.115 |
|  |  | Minimum | 16.9 | p<0.001 |
|  |  | 10 Percentile | 18.8 | p<0.001 |
|  |  | 90 Percentile | 10.9 | p<0.001 |
|  |  | Maximum | 19.3 | p<0.001 |
|  |  | Mean | 10.9 | p<0.001 |
|  |  | Median | 7.48 | 0.00632 |
|  |  | Range | 9.99 | 0.00160 |
|  |  | Interquartile Range | 2.61 | 0.106 |
|  |  | Root Mean Squared | 10.9 | p<0.001 |
|  |  | Standard Deviation | 1.67 | 0.196 |
|  |  | Skewness | 14.6 | p<0.001 |
|  |  | Kurtosis | 1.52 | 0.218 |
|  |  | Uniformity | 7.42 | 0.00651 |
|  | Shape 2D | Perimeter | 11.9 | p<0.001 |
|  |  | Perimeter Surface Ratio | 11.8 | p<0.001 |
|  |  | Sphericity | 17.4 | p<0.001 |
|  |  | Spherical Disproportion | 16.3 | p<0.001 |
|  |  | Maximum Diameter | 3.74 | 0.0532 |
|  |  | Major Axis Length | 6.63 | 0.0101 |
|  |  | Minor Axis Length | 2.43 | 0.119 |
|  |  | Elongation | 4.54 | 0.0332 |
|  | GLCM | Contrast | 3.33 | 0.0683 |
|  |  | Difference Entropy | 1.53 | 0.217 |
|  |  | Difference Variance | 9.02 | 0.00271 |
|  |  | Joint Energy | 9.50 | 0.00208 |
|  |  | Joint Entropy | 4.30 | 0.0382 |
|  |  | Informational Measure of Correlation 2 | 13.7 | p<0.001 |
|  |  | Maximal Correlation Coefficient | 9.45 | 0.00215 |
|  |  | Inverse Difference Moment | 10.5 | 0.00122 |
|  |  | Inverse Difference Normalized | 15.6 | p<0.001 |
|  |  | Maximum Probability | 6.82 | 0.00911 |
|  |  | Sum Entropy | 2.33 | 0.127 |
|  | GLSZM | Large Area Emphasis | 7.39 | 0.00662 |
|  |  | Gray Level Nonuniformity | 1.65 | 0.199 |
|  |  | Gray Level Nonuniformity Normalized | 14.9 | p<0.001 |
|  |  | Gray Level Variance | 1.44 | 0.231 |
|  |  | Zone Variance | 7.40 | 0.00660 |
|  |  | Zone Entropy | 14.4 | p<0.001 |
|  |  | Low Gray Level Zone Emphasis | 1.67 | 0.196 |
|  |  | High Gray Level Zone Emphasis | 1.39 | 0.239 |
|  |  | Small Area High Gray Level Emphasis | 1.50 | 0.221 |
|  |  | Large Area Low Gray Level Emphasis | 7.08 | 0.00788 |
|  | GLRLM | Short Run Emphasis | 1.74 | 0.187 |
|  |  | Long Run Emphasis | 6.85 | 0.00893 |
|  |  | Gray Level Nonuniformity | 2.03 | 0.154 |
|  |  | Gray Level Nonuniformity Normalized | 8.90 | 0.00288 |
|  |  | Run Percentage | 2.11 | 0.147 |
|  |  | Run Entropy | 3.79 | 0.0517 |
|  |  | Run Variance | 7.17 | 0.00751 |
|  |  | Low Gray Level Run Emphasis | 2.32 | 0.128 |
|  |  | Long Run Low Gray Level Emphasis | 7.29 | 0.00700 |
|  | NGTDM | Busyness | 7.85 | 0.00513 |
|  |  | Complexity | 5.51 | 0.0191 |
|  | GLDM | Large Dependence Emphasis | 4.52 | 0.0337 |
|  |  | Gray Level Nonuniformity | 4.10 | 0.0431 |
|  |  | Dependence Non Uniformity Normalized | 1.07 | 0.301 |
|  |  | Dependence Variance | 1.16 | 0.281 |
|  |  | Dependence Entropy | 8.96 | 0.00280 |
|  |  | Low Gray Level Emphasis | 2.56 | 0.109 |
|  |  | Small Dependence High Gray Level Emphasis | 1.08 | 0.300 |
|  |  | Large Dependence Low Gray Level Emphasis | 7.57 | 0.00601 |

Abbreviations: ET: enhancing tumor; NCR: tumor core; ED: peritumoral edematous/infiltrated tissue; GLCM: gray-level co-occurrence matrix; GLSZM: gray-level size zone matrix; GLRLM: Gray-level run length matrix; NGTDM: neighboring gray-tone difference matrix; GLDM: gray-level dependence matrix.

**Supplementary Table 3.** Radiomic features selected from ED of contrast-enhanced T1-weighted image

| Area | Feature class | Feature | F-value | p-value |
| --- | --- | --- | --- | --- |
| ED  (contrast-enhanced  T1-weighted  image) | First Order | pixels T1 edema | 6.65 | 0.0100 |
|  |  | Energy | 12.7 | p<0.001 |
|  |  | Total Energy | 12.7 | p<0.001 |
|  |  | Entropy | 8.03 | 0.00465 |
|  |  | Minimum | 30.8 | p<0.001 |
|  |  | 10 Percentile | 19.6 | p<0.001 |
|  |  | 90 Percentile | 22.2 | p<0.001 |
|  |  | Maximum | 5.49 | 0.0193 |
|  |  | Mean | 20.3 | p<0.001 |
|  |  | Median | 18.0 | p<0.001 |
|  |  | Interquartile Range | 5.56 | 0.0185 |
|  |  | Mean Absolute Deviation | 5.54 | 0.0187 |
|  |  | Robust Mean Absolute Deviation | 5.84 | 0.0158 |
|  |  | Root Mean Squared | 20.5 | p<0.001 |
|  |  | Skewness | 3.74 | 0.0535 |
|  |  | Uniformity | 11.4 | p<0.001 |
|  | Shape 2D | Mesh Surface | 6.64 | 0.0100 |
|  |  | Pixel Surface | 6.64 | 0.0100 |
|  |  | Perimeter | 53.1 | p<0.001 |
|  |  | Perimeter Surface Ratio | 4.17 | 0.0413 |
|  |  | Sphericity | 71.3 | p<0.001 |
|  |  | Spherical Disproportion | 63.5 | p<0.001 |
|  |  | Maximum Diameter | 23.4 | p<0.001 |
|  |  | Major Axis Length | 101.7 | p<0.001 |
|  |  | Minor Axis Length | 12.9 | p<0.001 |
|  |  | Elongation | 34.8 | p<0.001 |
|  | GLCM | Difference Entropy | 7.24 | 0.00720 |
|  |  | Joint Energy | 10.5 | 0.00122 |
|  |  | Joint Entropy | 5.62 | 0.0179 |
|  |  | Informational Measure of Correlation 2 | 6.69 | 0.00977 |
|  |  | Inverse Difference Moment | 12.0 | p<0.001 |
|  |  | Inverse Difference | 10.8 | 0.00104 |
|  |  | Inverse Variance | 5.85 | 0.0157 |
|  |  | Maximum Probability | 10.2 | 0.00141 |
|  |  | Sum Entropy | 6.28 | 0.0123 |
|  | GLSZM | Small Area Emphasis | 6.88 | 0.00879 |
|  |  | Large Area Emphasis | 6.22 | 0.0127 |
|  |  | Gray Level Nonuniformity | 14.6 | p<0.001 |
|  |  | Gray Level Nonuniformity Normalized | 12.4 | p<0.001 |
|  |  | Size Zone None Uniformity Normalized | 4.82 | 0.0282 |
|  |  | Zone Percentage | 9.26 | 0.00238 |
|  |  | Zone Variance | 6.15 | 0.0132 |
|  |  | Low Gray Level Zone Emphasis | 6.57 | 0.0104 |
|  |  | Large Area Low Gray Level Emphasis | 6.22 | 0.0127 |
|  |  | Large Area High Gray Level Emphasis | 7.78 | 0.00533 |
|  | GLRLM | Short Run Emphasis | 12.0 | p<0.001 |
|  |  | Long Run Emphasis | 10.1 | 0.00149 |
|  |  | Gray Level Nonuniformity | 26.1 | p<0.001 |
|  |  | Gray Level Nonuniformity Normalized | 11.7 | p<0.001 |
|  |  | Run Length Nonuniformity Normalized | 10.7 | 0.00111 |
|  |  | Run Percentage | 12.5 | p<0.001 |
|  |  | Run Entropy | 4.00 | 0.0457 |
|  |  | Run Variance | 9.67 | 0.00191 |
|  |  | Low Gray Level Run Emphasis | 5.52 | 0.0189 |
|  |  | Long Run Low Gray Level Emphasis | 8.11 | 0.00445 |
|  | NGTDM | Busyness | 13.1 | p<0.001 |
|  |  | Coarseness | 4.53 | 0.0334 |
|  | GLDM | Small Dependence Emphasis | 7.16 | 0.00754 |
|  |  | Large Dependence Emphasis | 12.4 | p<0.001 |
|  |  | Gray Level Nonuniformity | 19.1 | p<0.001 |
|  |  | Dependence Nonuniformity Normalized | 4.52 | 0.0336 |
|  |  | Dependence Variance | 12.8 | p<0.001 |
|  |  | Low Gray Level Emphasis | 5.06 | 0.0246 |
|  |  | Large Dependence Low Gray Level Emphasis | 8.62 | 0.00338 |

Abbreviations: ET: enhancing tumor; NCR: tumor core; ED: peritumoral edematous/infiltrated tissue; GLCM: gray-level co-occurrence matrix; GLSZM: gray-level size zone matrix; GLRLM: gray-level run-length matrix; NGTDM: neighboring gray-tone difference matrix; GLDM: gray-level dependence matrix.

**Supplementary Table 4.** Radiomic features selected from ET of T2-weighted image

| Area | Feature class | Feature | F-value | p-value |
| --- | --- | --- | --- | --- |
| ET  (T2-weighted images) | First Order | pixels T2emhance | 55.9 | p<0.001 |
|  |  | Entropy | 58.9 | p<0.001 |
|  |  | Minimum | 26.3 | p<0.001 |
|  |  | 90 Percentile | 43.3 | p<0.001 |
|  |  | Maximum | 33.1 | p<0.001 |
|  |  | Mean | 27.7 | p<0.001 |
|  |  | Interquartile Range | 74.8 | p<0.001 |
|  |  | Mean Absolute Deviation | 69.4 | p<0.001 |
|  |  | Robust Mean Absolute Deviation | 75.8 | p<0.001 |
|  |  | Root Mean Squared | 29.8 | p<0.001 |
|  |  | Standard Deviation | 60.8 | p<0.001 |
|  |  | Kurtosis | 25.0 | p<0.001 |
|  |  | Variance | 49.0 | p<0.001 |
|  |  | Uniformity | 42.8 | p<0.001 |
|  | Shape 2D | Mesh Surface | 55.5 | p<0.001 |
|  |  | Pixel Surface | 55.9 | p<0.001 |
|  |  | Perimeter Surface Ratio | 35.9 | p<0.001 |
|  | GLCM | Cluster Prominence | 25.2 | p<0.001 |
|  |  | Cluster Shade | 25.3 | p<0.001 |
|  |  | Cluster Tendency | 48.9 | p<0.001 |
|  |  | Contrast | 46.3 | p<0.001 |
|  |  | Difference Average | 72.1 | p<0.001 |
|  |  | Difference Entropy | 77.9 | p<0.001 |
|  |  | Difference Variance | 38.6 | p<0.001 |
|  |  | Joint Energy | 30.6 | p<0.001 |
|  |  | Joint Entropy | 41.0 | p<0.001 |
|  |  | Informational Measure of Correlation 1 | 39.0 | p<0.001 |
|  |  | Informational Measure of Correlation 2 | 34.2 | p<0.001 |
|  |  | Inverse Difference Moment | 74.5 | p<0.001 |
|  |  | Inverse Difference Moment Normalized | 52.7 | p<0.001 |
|  |  | Inverse Difference | 76.7 | p<0.001 |
|  |  | Inverse Difference Normalized | 61.8 | p<0.001 |
|  |  | Inverse Variance | 82.7 | p<0.001 |
|  |  | Sum Entropy | 48.2 | p<0.001 |
|  |  | Sum Squares | 49.3 | p<0.001 |
|  | GLSZM | Small Area Emphasis | 73.6 | p<0.001 |
|  |  | Large Area Emphasis | 36.1 | p<0.001 |
|  |  | Gray Level Nonuniformity | 86.0 | p<0.001 |
|  |  | Gray Level Nonuniformity Normalized | 42.3 | p<0.001 |
|  |  | Size Zone Nonuniformity Normalized | 75.7 | p<0.001 |
|  |  | Zone Percentage | 75.1 | p<0.001 |
|  |  | Gray Level Variance | 45.5 | p<0.001 |
|  |  | Zone Variance | 30.0 | p<0.001 |
|  |  | Large Area High Gray Level Emphasis | 31.1 | p<0.001 |
|  | GLRLM | Short Run Emphasis | 76.4 | p<0.001 |
|  |  | Long Run Emphasis | 59.7 | p<0.001 |
|  |  | Gray Level Nonuniformity | 98.0 | p<0.001 |
|  |  | Gray Level Nonuniformity Normalized | 43.4 | p<0.001 |
|  |  | Run Length Nonuniformity | 78.5 | p<0.001 |
|  |  | Run Length Nonuniformity Normalized | 70.7 | p<0.001 |
|  |  | Run Percentage | 48.3 | p<0.001 |
|  |  | Gray Level Variance | 37.0 | p<0.001 |
|  |  | Run Entropy | 50.6 | p<0.001 |
|  |  | Run Variance | 76.4 | p<0.001 |
|  | NGTDM | Busyness | 26.0 | p<0.001 |
|  |  | Coarseness | 35.5 | p<0.001 |
|  |  | Complexity | 31.9 | p<0.001 |
|  |  | Contrast | 67.7 | p<0.001 |
|  |  | Strength | 37.8 | p<0.001 |
|  | GLDM | Small Dependence Emphasis | 76.6 | p<0.001 |
|  |  | Large Dependence Emphasis | 63.8 | p<0.001 |
|  |  | Gray Level Nonuniformity | 93.8 | p<0.001 |
|  |  | Dependence Nonuniformity Normalized | 74.5 | p<0.001 |
|  |  | Gray Level Variance | 49.0 | p<0.001 |
|  |  | Dependence Variance | 48.8 | p<0.001 |

Abbreviations: ET: enhancing tumor; NCR: tumor core; ED: peritumoral edematous/infiltrated tissue; GLCM: gray-level co-occurrence matrix; GLSZM: gray-level size zone matrix; GLRLM: gray-level run-length matrix; NGTDM: neighboring gray-tone difference matrix; GLDM: gray-level dependence matrix.

**Supplementary Table 5.** Radiomic features selected from NCR of T2-weighted image

| Area | Feature class | Feature | F-value | p-value |
| --- | --- | --- | --- | --- |
| NCR  (T2-weighted images) | First Order | Energy | 40.0 | p<0.001 |
|  |  | Total Energy | 40.0 | p<0.001 |
|  |  | Entropy | 15.9 | p<0.001 |
|  |  | Minimum | 17.4 | p<0.001 |
|  |  | 10 Percentile | 35.7 | p<0.001 |
|  |  | 90 Percentile | 56.9 | p<0.001 |
|  |  | Maximum | 44.7 | p<0.001 |
|  |  | Mean | 54.0 | p<0.001 |
|  |  | Median | 55.5 | p<0.001 |
|  |  | Range | 27.3 | p<0.001 |
|  |  | Interquartile Range | 8.90 | 0.00290 |
|  |  | Mean Absolute Deviation | 20.4 | p<0.001 |
|  |  | Robust Mean Absolute Deviation | 11.0 | p<0.001 |
|  |  | Root Mean Squared | 55.4 | p<0.001 |
|  |  | Standard Deviation | 28.6 | p<0.001 |
|  |  | Skewness | 28.3 | p<0.001 |
|  |  | Kurtosis | 8.80 | 0.00303 |
|  |  | Variance | 27.4 | p<0.001 |
|  | Shape 2D | Perimeter | 11.9 | p<0.001 |
|  |  | Perimeter Surface Ratio | 11.8 | p<0.001 |
|  |  | Sphericity | 17.4 | p<0.001 |
|  |  | Spherical Disproportion | 16.3 | p<0.001 |
|  | GLCM | Autocorrelation | 36.6 | p<0.001 |
|  |  | Joint Average | 43.0 | p<0.001 |
|  |  | Cluster Prominence | 22.3 | p<0.001 |
|  |  | Cluster Shade | 19.5 | p<0.001 |
|  |  | Cluster Tendency | 23.9 | p<0.001 |
|  |  | Correlation | 32.2 | p<0.001 |
|  |  | Difference Variance | 15.0 | p<0.001 |
|  |  | Informational Measure of Correlation 1 | 37.5 | p<0.001 |
|  |  | Informational Measure of Correlation 2 | 40.0 | p<0.001 |
|  |  | Maximal Correlation Coefficient | 63.1 | p<0.001 |
|  |  | Inverse Difference Moment Normalized | 30.4 | p<0.001 |
|  |  | Inverse Difference Normalized | 44.1 | p<0.001 |
|  |  | Sum Average | 43.0 | p<0.001 |
|  |  | Sum Entropy | 12.8 | p<0.001 |
|  |  | Sum Squares | 23.4 | p<0.001 |
|  | GLSZM | Large Area Emphasis | 14.9 | p<0.001 |
|  |  | Gray Level Nonuniformity | 9.70 | 0.00191 |
|  |  | Gray Level Nonuniformity Normalized | 16.3 | p<0.001 |
|  |  | Gray Level Variance | 29.6 | p<0.001 |
|  |  | Zone Variance | 16.2 | p<0.001 |
|  |  | Zone Entropy | 38.0 | p<0.001 |
|  |  | Low Gray Level Zone Emphasis | 10.4 | 0.00129 |
|  |  | High Gray Level Zone Emphasis | 35.6 | p<0.001 |
|  |  | Small Area Low Gray Level Emphasis | 11.5 | p<0.001 |
|  |  | Small Area High Gray Level Emphasis | 32.1 | p<0.001 |
|  |  | Large Area Low Gray Level Emphasis | 13.1 | p<0.001 |
|  |  | Large Area High Gray Level Emphasis | 11.5 | p<0.001 |
|  | GLRLM | Gray Level Nonuniformity Normalized | 9.37 | 0.00224 |
|  |  | Gray Level Variance | 27.3 | p<0.001 |
|  |  | Run Entropy | 29.8 | p<0.001 |
|  |  | High Gray Level Run Emphasis | 36.2 | p<0.001 |
|  |  | Short Run High Gray Level Emphasis | 35.5 | p<0.001 |
|  |  | Long Run High Gray Level Emphasis | 23.8 | p<0.001 |
|  | NGTDM | Busyness | 29.7 | p<0.001 |
|  |  | Complexity | 22.2 | p<0.001 |
|  |  | Strength | 31.2 | p<0.001 |
|  | GLDM | Gray Level Variance | 27.4 | p<0.001 |
|  |  | Dependence Entropy | 44.7 | p<0.001 |
|  |  | High Gray Level Emphasis | 36.2 | p<0.001 |
|  |  | Small Dependence Low Gray Level Emphasis | 11.6 | p<0.001 |
|  |  | Small Dependence High Gray Level Emphasis | 29.5 | p<0.001 |
|  |  | Large Dependence High Gray Level Emphasis | 17.6 | p<0.001 |

Abbreviations: ET: enhancing tumor; NCR: tumor core; ED: peritumoral edematous/infiltrated tissue; GLCM: gray-level co-occurrence matrix; GLSZM: gray-level size zone matrix; GLRLM: gray-level run-length matrix; NGTDM: neighboring gray-tone difference matrix; GLDM: gray-level dependence matrix.

**Supplementary Table 6.** Radiomic features selected from ED of T2-weighted image

| Area | Feature class | Feature | F-value | p-value |
| --- | --- | --- | --- | --- |
| ED  (T2-weighted images) | First Order | pixels T2edema | 6.65 | 0.0100 |
|  |  | Energy | 3.41 | 0.0651 |
|  |  | Total Energy | 3.41 | 0.0651 |
|  |  | Minimum | 50.9 | p<0.001 |
|  |  | 10 Percentile | 31.5 | p<0.001 |
|  |  | 90 Percentile | 15.1 | p<0.001 |
|  |  | Maximum | 1.77 | 0.184 |
|  |  | Mean | 20.9 | p<0.001 |
|  |  | Median | 22.4 | p<0.001 |
|  |  | Range | 2.06 | 0.152 |
|  |  | Interquartile Range | 1.08 | 0.299 |
|  |  | Mean Absolute Deviation | 1.52 | 0.218 |
|  |  | Robust Mean Absolute Deviation | 1.58 | 0.209 |
|  |  | Root Mean Squared | 19.4 | p<0.001 |
|  |  | Standard Deviation | 8.59 | 0.00343 |
|  |  | Skewness | 1.35 | 0.246 |
|  |  | Kurtosis | 4.08 | 0.0436 |
|  |  | Variance | 1.28 | 0.259 |
|  |  | Uniformity | 6.65 | 0.0100 |
|  | Shape 2D | Mesh Surface | 6.64 | 0.0100 |
|  |  | Pixel Surface | 6.65 | 0.0100 |
|  |  | Perimeter | 53.1 | p<0.001 |
|  |  | Perimeter Surface Ratio | 4.17 | 0.0413 |
|  |  | Sphericity | 71.3 | p<0.001 |
|  |  | Spherical Disproportion | 63.5 | p<0.001 |
|  |  | Maximum Diameter | 23.4 | p<0.001 |
|  |  | Major Axis Length | 101.7 | p<0.001 |
|  |  | Minor Axis Length | 12.9 | p<0.001 |
|  |  | Elongation | 34.8 | p<0.001 |
|  | GLCM | Cluster Prominence | 10.5 | 0.00122 |
|  |  | Cluster Shade | 6.92 | 0.00860 |
|  |  | Cluster Tendency | 5.25 | 0.0221 |
|  |  | Contrast | 5.48 | 0.0194 |
|  |  | Correlation | 4.43 | 0.0354 |
|  |  | Difference Variance | 3.90 | 0.0485 |
|  |  | Joint Energy | 2.21 | 0.138 |
|  |  | Informational Measure of Correlation 1 | 15.8 | p<0.001 |
|  |  | Informational Measure of Correlation 2 | 3.24 | 0.0720 |
|  |  | Maximal Correlation Coefficient | 4.83 | 0.0282 |
|  |  | Inverse Variance | 1.87 | 0.172 |
|  |  | Sum Squares | 5.37 | 0.0206 |
|  | GLSZM | Small Area Emphasis | 1.19 | 0.276 |
|  |  | Large Area Emphasis | 3.54 | 0.0600 |
|  |  | Gray Level Nonuniformity | 16.3 | p<0.001 |
|  |  | Size Zone Nonuniformity | 13.2 | p<0.001 |
|  |  | Gray Level Variance | 2.74 | 0.0980 |
|  |  | Zone Variance | 3.72 | 0.0539 |
|  |  | Low Gray Level Zone Emphasis | 1.55 | 0.214 |
|  |  | Large Area Low Gray Level Emphasis | 6.68 | 0.00984 |
|  |  | Large Area High Gray Level Emphasis | 9.44 | 0.00216 |
|  | GLRLM | Gray Level Nonuniformity | 8.66 | 0.00330 |
|  |  | Gray Level Nonuniformity Normalized | 1.20 | 0.274 |
|  |  | Run Length Nonuniformity | 10.4 | 0.00131 |
|  |  | Gray Level Variance | 3.83 | 0.0506 |
|  |  | Low Gray Level Run Emphasis | 1.64 | 0.201 |
|  |  | Long Run Low Gray Level Emphasis | 4.88 | 0.0273 |
|  | NGTDM | Busyness | 8.45 | 0.00370 |
|  |  | Coarseness | 12.3 | p<0.001 |
|  |  | Complexity | 3.23 | 0.0726 |
|  |  | Contrast | 2.31 | 0.129 |
|  | GLDM | Gray Level Nonuniformity | 7.29 | 0.00699 |
|  |  | Dependence Nonuniformity | 11.0 | p<0.001 |
|  |  | Gray Level Variance | 4.09 | 0.0434 |
|  |  | Low Gray Level Emphasis | 1.67 | 0.197 |
|  |  | Large Dependence Low Gray Level Emphasis | 5.46 | 0.0196 |

Abbreviations: ET: enhancing tumor; NCR: tumor core; ED: peritumoral edematous/infiltrated tissue; GLCM: gray-level co-occurrence matrix; GLSZM: gray-level size zone matrix; GLRLM: gray-level run-length matrix; NGTDM: neighboring gray-tone difference matrix; GLDM: gray-level dependence matrix.

**Appendix 4** Detailed explanation for vViT architecture

**Supplemental Figure 2** shows an architectural overview of the vViT. The vViT receives a 1-dimensional input sequence which is constructed by merging all input (demographics, radiomic features, and medical images). Before merging, all arrays are converted to 1-dimensional arrays. The input sequence undergoes splitting procedures according to the split-sequence. Using split-sequence. we input demographics, radiomic features and 2-dimensional MR images to vViT by converting them to 1-dimensional arrays. We added a class token to the input following the ViT implementation [3]. The smaller split sequences are input into the transformer encoder after the splitting procedure [4]. The transformer encoder comprises alternating layers of multiheaded self-attention and multilayer perceptron (MLP) blocks. Layernorm is applied before every block, and residual connections after every block [3]. We implemented MLP using two layers with a Gaussian Error Linear Unit (GELU) non-linearity. Specifically, we set the patch dimension, head dimension, number of heads, MLP dimension, and depth to 64, 64, 4, 64, and 4, respectively. The classification head is implemented by an MLP with four hidden layers and a SoftMax function. Hence, vViT contains an arbitrary number of sectors that receive sequences of different lengths. The probability tensor that contained output from each sector was integrated by voting into the total model output. The respective probabilities of MGMT-methylated and unmethylated in all sectors in this integration were averaged. The total model output was determined using averaged probabilities. The output of each sector in vViT can be derived from the probability tensor. The present study defined each sector according to the property of the input; for example, the class token sector, demographic sector, radiomic sector, and CE-T1WI sector.

**Supplementary Figure 2.** Scheme of the variable vision transformer (vViT). The vViT receives input and split-sequence $\left\{ a_{n} \right\}, 1\leq n\leq N.$ The length of the input sequence is $\sum_{i=1}^{N} a_{i}$. The split sequences are then input to each sector, with the length of $a_{1}, a_{2}, \cdots, a_{N}$. Each sequence is input into the transformer encoder after concatenating the class token. L denotes the depth of transformer encoder. Inspired by the original ViT encoder, the transformer is composed of a norm layer (Norm), multi-head attention layer, and multilayer perceptron (MLP). Finally, MLP head and voting procedures are performed to the probability tensor, and the total model output is obtained.

***
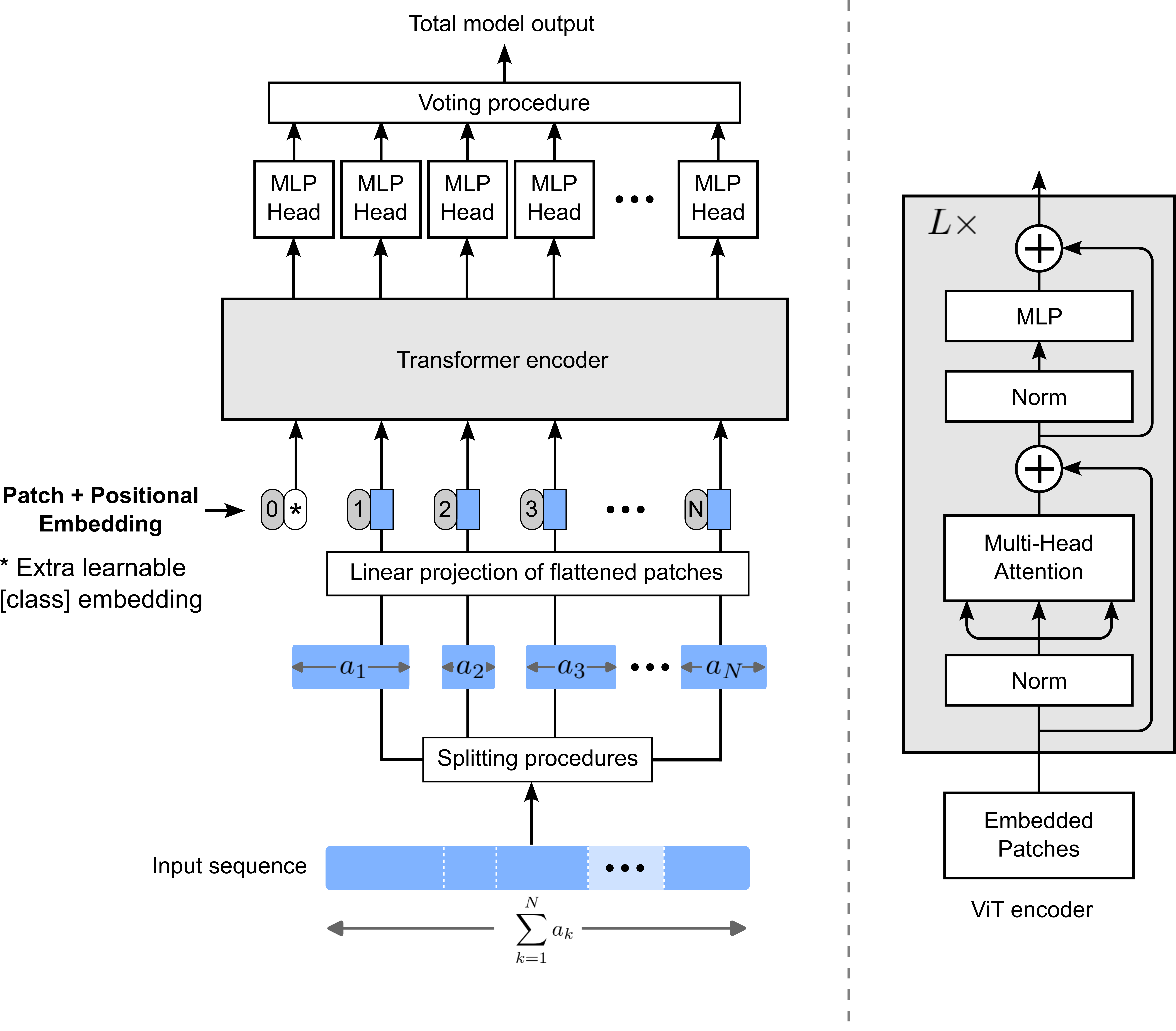
***

***Terminology list for vViT***

| Class token | In ViT, the output of this token is then transformed into a class prediction [3]. Although the prediction of each sector can be derived in vViT, we used class token for two reasons: first, to stay as close as possible to the original transformer model, and second, to make the number of sectors odd for voting. |
| --- | --- |
| Classification head | The classification head makes the output from the transformer encoder to prediction. |
| Depth | The depth of the transformer encoder. In **Supplementary Figure 2,** L denotes the depth. |
| Input sequence | Input sequence was composed by merging all inputs (demographics, radiomic features, and medical images) after transforming into 1-dimensional array. |
| Multiheaded self-attention | In the standard self-attention, query, key, and value are calculated to make predictions [3]. Multihead self-attention is an extension of self-attention in which we run multiple self-attention operations, called “heads”, in parallel, and project their concatenated outputs [3]. |
| Patch dimension | The dimension of output from the transformer encoder. |
| Positional embedding | Positional embedding encodes spatial information using trigonometric functions in the original ViT [3]. In vViT, positional embedding provides information on what each sector receives. |
| Probability tensor | The output of each sector is ranged from 0 to 1 and can be interpreted as probability. The output is derived in the form of a tensor: we call this tensor a probability tensor. |
| Sector | The unit composed of a smaller split sequence, transformer, and multilayer perceptron is called a sector. |
| Smaller split sequences | The sequences after splitting the input sequence according to the split sequence. |
| Split sequence | The split sequence determines how to split an input sequence. |
| Splitting procedure | The procedure of splitting the input sequence according to a split sequence. |
| Transformer | In the original paper [5], the transformer is an architecture that is composed of stacked self-attention and point-wise, fully connected layers for both the encoder and decoder. |
| Voting | By voting, the majority of predictions from sectors are determined. So, the number of sectors should be odd. |

***Terminology list for deep learning***

| Adam optimizer | An algorithm for first-order gradient-based optimization of stochastic objective functions. |
| --- | --- |
| Binary cross entropy | A loss function based on the following equation which evaluates the difference between calculated probability and ground truth in binary classification.  $Binary cross entropy=-\frac{1}{N}\sum_{i=1}^{N} y_{i}\log\left( p_{i} \right)+(1-y_{i})log(1-p_{i})$ |
| Gaussian Error Linear Unit | The Gaussian Error Linear Unit, or GELU, is an activation function. |
| Hidden layer | The layer between the input and output layers. |
| Layernorm | Layer normalization over a mini-batch of inputs. |
| Multilayer perceptron | Multilayer perceptron is a supplement of a feed-forward neural network. It consists of three types of layers; the input layer, the output layer, and the hidden layer. |
| Residual connections | Residual Connections are a type of skip-connection that learns residual functions with reference to the layer inputs, instead of learning unreferenced functions. |
| SoftMax function | SoftMax function converts a vector into a probability distribution. Using this function we can interpret the output from vViT as probability. |

**References**

1. Liu F, Ning Z, Liu Y, Liu D, Tian J, Luo H, et al. Development and validation of a radiomics signature for clinically significant portal hypertension in cirrhosis (CHESS1701): a prospective multicenter study. *EBioMedicine* 2018;36:151-158

2. van Griethuysen JJM, Fedorov A, Parmar C, Hosny A, Aucoin N, Narayan V, et al. Computational Radiomics System to Decode the Radiographic Phenotype. *Cancer Res* 2017;77:e104-e107

3. Dosovitskiy A, Beyer L, Kolesnikov A, Weissenborn D, Zhai X, Unterthiner T, et al. An Image is Worth 16x16 Words: Transformers for Image Recognition at Scale 2020

4. Usuzaki T. Splitting expands the application range of Vision Transformer -- variable Vision Transformer (vViT) 2022

5. Vaswani A, Shazeer N, Parmar N, Uszkoreit J, Jones L, Gomez AN, et al. Attention Is All You Need 2017
